# Social identity coupling model in university students in high-level sports teams: based on grounded theory and fsQCA

**DOI:** 10.3389/fpsyg.2026.1971252

**Published:** 2026-10-05

**Authors:** Huabin Hang, Jiangping Fu, Cairong Wu, Yayangzi Sun, Zhi Sun

**Affiliations:** 1 School of Physical Education, Nanjing University of Technology, Nanjing, Jiangsu, China; 2 Nantong Institute of Technology, Nantong, Jiangsu, China; 3 Jinling Institute of Technology, Nanjing, Jiangsu, China; 4 Gannan Health Vocational College, Ganzhou, Jiangxi, China; 5 Jiangsu Open University, Nanjing, Jiangsu, China

**Keywords:** fuzzy-set qualitative comparative analysis, grounded theory, high-level sports teams, inclusive leadership, Physical literacy, sense of belonging, social identity coupling model

## Abstract

**Background:**

University students from high-level sports teams in China assume dual roles as formal students and elite athletes. This study develops a localized social identity coupling model to address the theoretical and empirical gaps in the existing literature.

**Method:**

Based on social identity theory, this study employs a mixed-method research paradigm integrating grounded theory and fuzzy-set qualitative comparative analysis (fsQCA). The sample comprises 36 university students from high-level sports teams in China recruited from six universities. Semi-structured interviews and three-stage coding procedures were performed to extract core conceptual dimensions, thereby constructing a specialized social identity coupling model that accommodates the dual-role characteristics of student-athletes. The fsQCA technique was implemented to systematically evaluate configurational mechanisms, structural robustness, empirical reliability, and hierarchical bottleneck characteristics of the proposed model.

**Results:**

This study establishes a five-dimensional coupling model of social identity for university students from high-level sports teams in China, encompassing: (1) a dual-dimensional perceived physical literacy kernel; (2) a dual-dimensional sense of belonging construction logic; (3) a single-dimensional inclusive leadership openness attribute; (4) a dual identity categorization conduction path; and (5) an inter-group comparison output path. The model achieves robust empirical fitness, as validated through theoretical saturation tests, configurational robustness analysis, reliability examination, and bottleneck level estimation using necessary condition analysis. Quantitative findings reveal that social identity is not determined by any single necessary condition but is collectively shaped by asymmetric multi-factor configurations. Two valid configurations conducive to high social identity are identified (overall consistency 0.89; coverage 0.63), among which inclusive leadership serves as the core cross-configuration condition. By contrast, two configurational paths corresponding to non-high social identity are extracted (overall consistency 0.77; coverage 0.69), and the absence of dual identity categorization constitutes the primary inhibitory factor. Bottleneck level analysis further confirms rigid minimum threshold constraints for all antecedents at high identity levels (≥50%), demonstrating distinct configurational functions of outcome promotion and risk prevention across variables.

**Conclusion:**

This study innovatively constructs and empirically validates a localized social identity coupling model targeted at university students from high-level sports teams in China.

## 1 Introduction

With unique embodied cognitive attributes (Fu et al., 2025a), physical literacy has become the logical starting point for integrating physical and mental well-being with sustainable career development among university students from high-level sports teams in China (Fu et al., 2025b). As core talent groups cultivated within elite sports contexts (Fu et al., 2026), the development of physical literacy among university students from high-level sports teams in China is not merely a process of individual ability improvement (Diao et al., 2024), but serves as an effective entry point for dual career preparation encompassing physical activity and perceptual physical literacy (Yan et al., 2022). Early studies have confirmed that career-oriented perceived physical literacy facilitates the deep organizational embedding and social identity development of university students from high-level sports teams in China through dynamic practical engagement (Luo et al., 2022). The International Physical Literacy Association (IPLA) has proposed the adaptability of the three-dimensional physical literacy framework, including motivation and cognition, physical competence, and knowledge and understanding (International Physical Literacy Association and Grace, 2026). This theoretical framework is applicable to youth populations (Ma et al., 2020), and provides a core psychological analytical paradigm for subsequent subgroup research targeting university student-athletes in high-level sports teams (Sum et al., 2018). Nevertheless, from an organizational psychology perspective (Cui et al., 2026), empirical evidence clarifying the positive predictive effect of physical literacy on the whole-life-cycle healthy behaviors of university students from high-level sports teams in China within previous dynamic educational and organizational contexts remains insufficient (Ma et al., 2020; Sum et al., 2018; Cui et al., 2026; Sum et al., 2016; Hu and Wang, 2025), constituting a critical research gap. Although university students from high-level sports teams in China possess favorable cognitive and psychological foundations for dual perceptions of physical health awareness and physical activity (Hu and Wang, 2025), supplementary psychological construction based on social identity theory is still required to enrich interdisciplinary research at the intersection of organizational and educational psychology.

Classic educational psychology research has systematically elaborated the formative mechanisms of youth belongingness, verifying that a sense of belonging serves as a fundamental psychological basis for social integration and mental health maintenance (Sotardi and Rudolf, 2026). From an organizational psychology perspective, belongingness reflects positive interactive psychological bonds between individual student-athletes and high-level sports team organizations (Allen et al., 2021), and characterizes the organizational dynamics of high-performance athletic teams (Allen et al., 2021). Within the analytical framework of social identity theory, the formation of belongingness among university students from high-level sports teams in China is a complex psychological mechanism deeply bound to group categorization, in-group preference, and value internalization, rather than a simple emotional attachment process (Ge et al., 2026). Context-specific research on university sports has further validated that sports group interaction and informal leadership structures positively facilitate the development of belongingness among university students from high-level sports teams in China (Slaten et al., 2016). Additionally, targeted studies on special populations have confirmed that identity conflict directly undermines individual group belongingness (Antonsich, 2010), establishing a theoretical reference for specialized analysis of athlete groups (Trajković et al., 2023). Emerging positive psychology research has further expanded the research boundary of belongingness by exploring its dual promotional effects on academic adaptation and athletic performance among athletes (Rokach and Goldberg, 2021; Rippon, 2023). Although existing studies have developed mature measurement dimensions for belongingness applicable to university students from high-level sports teams in China, empirical exploration of the linkage mechanism between belongingness and perceived physical literacy remains absent (Grisoni, 2022). This research gap restricts the design of educational strategies and practical schemes for the coordinated development of dual identities among student-athletes in sports team contexts.

Classic organizational behavior research has systematically confirmed the positive empowering effects of inclusive leadership on identity recognition and work engagement among employees with diverse backgrounds (Al-Atwi and Al-Hassani, 2021). In the dual-career preparation context (King et al., 2024), perceived inclusive leadership serves as a core mediating variable linking social identity construction, belongingness formation, and physical literacy development for university students from high-level sports teams in China (Yoon and Hong, 2026). Multiple studies on inclusive leadership have verified the critical value of fair evaluation and diverse respect for the organizational integration of members in special groups (Nguyen et al., 2024; Li et al., 2025). Early explorations in sports team research have further demonstrated that inclusive leadership effectively reduces athletes’ identity conflict perception, constructing a core theoretical framework for high-level sports team research (Cheng et al., 2022). Recent frontier studies have embedded social identity theory deeply into university sports organizational research, exploring the activation paths of inclusive leadership on social identity among elite athletes (Chen et al., 2024). However, existing research on sports organizational inclusiveness predominantly focuses on professional sports teams, neglecting the unique dual-career preparation attributes of university students from high-level sports teams in China (Zhang et al., 2025). While mature measurement tools for inclusive leadership adapted to Chinese university high-level sports teams have been developed (Wu and Li, 2023), systematic empirical examination of the linkage mechanism among inclusive leadership, sense of belonging, and perceived physical literacy based on social identity theory remains lacking. This gap hinders the precise construction of multi-collaborative educational systems for university high-level sports teams (Qi et al., 2023).

Current research on university students from high-level sports teams in China is gradually transitioning from single-variable net effect analysis to exploration of complex multi-configurational causal mechanisms (Zhang and Li, 2022; Williams, 2023; Koo et al., 2025). The simultaneous integration of social identity theory (theoretical framework, Ai and Tanimoto, 2026), grounded theory (research method, Sun et al., 2026), and fsQCA (analytical tool, Pappas and Woodside, 2021) may represent a core pathway to unpack the black box of interrelated multiple psychological factors among university students from high-level sports teams in China (Harrison et al., 2024). Individually, social identity theory has achieved localized adaptation in sports psychology research (Khan et al., 2023), providing a mature analytical framework for studies on athlete identity recognition (Ahmad et al., 2022) and group interaction (Jiang et al., 2022). Grounded theory enables in-depth qualitative coding of sports group psychological factors and systematic extraction of multi-dimensional conceptual connotations (Montes and Penzenstadler, 2026). FsQCA has been widely validated across social science disciplines for its unique advantages in identifying multi-condition concurrent relationships (Berg-Schlosser and De Meur, 2009). Whether social identity theory, grounded theory, or fsQCA, the three approaches have independently accumulated solid theoretical and empirical foundations. Ongoing academic attempts have integrated the three approaches sequentially: grounded theory calibrates core conceptual dimensions in localized contexts (Yang and Shi, 2026), social identity theory constructs holistic analytical frameworks (Araújo et al., 2025), and fsQCA identifies combinational influence paths of diverse factors. These integrated explorations have yielded significant analytical benefits in youth psychological research (Tabassum et al., 2023). Nevertheless, prominent research gaps remain. Existing studies on university students from high-level sports teams in China fail to achieve systematic integration of the three approaches, lacking grounded-theory-based localized dimension calibration and fsQCA-based identification of multi-concurrent paths among perceived physical literacy, sense of belonging, and inclusive leadership. This paradigmatic deficiency limits the practical transformation of research conclusions in this field.

Based on the aforementioned literature review, this study takes the dual-career role attributes of university students from high-level sports teams in China as the starting point and regards perceived physical literacy as the logical origin of perception-behavior theory, aiming to evaluate the organizational psychological structural characteristics of their perceived physical literacy, sense of belonging, and inclusive leadership. First, theoretically, this study constructs a social identity coupling model for the organizational psychology of university students from high-level sports teams in China based on grounded theory and social identity theory. Second, mechanistically, this study adopts the fsQCA algorithm to explore the path characteristics of the social identity coupling model for university students from high-level sports teams in China. Third, practically, based on the configurational path results, this study proposes practical strategies for the social identity coupling model of university students from high-level sports teams in China, and identifies the bottleneck level characteristics of the three antecedent variables (perceived physical literacy, sense of belonging, and inclusive leadership).

## 2 Literature review: social identity theory

Social identity theory is highly applicable to organizational and educational psychological research on university students from high-level sports teams in China (Ai and Tanimoto, 2026), primarily due to the precise logical matching between its classic “categorization-identification-comparison” framework (Harrison et al., 2024; Tajfel and Turner, 1979) and the dual attributes of this special group as members of semi-formal athletic organizations and campus student communities (Khan et al., 2023; Tajfel et al., 1971).

In the categorization stage, university students from high-level sports teams in China may spontaneously categorize themselves into two core social groups: elite athletes and university students, forming dual identity classification centered on athletic training goals, team honor, and academic learning scenarios, respectively, (Ahmad et al., 2022).

In the identification stage, they establish emotional bonds with athletic teams to obtain competitive identity belongingness while reinforcing student identity cognition in campus life, conforming to the core proposition of social identity theory that multiple social identities buffer single identity threats (Araújo et al., 2025; Turner et al., 1987).

In the comparison stage, individuals enhance collective self-esteem through in-group positive differentiation in athletic teams and construct positive self-images with dual advantages of sports expertise and academic pursuit through inter-group comparison with ordinary students on campus, which fully aligns with the mechanism of acquiring positive social identity via inter-group comparison (Jiang et al., 2022).

This integrated framework retains the complete connotation of traditional social identity theory while adapting to the dual-role characteristics of university students from high-level sports teams in China, providing solid theoretical support for interpreting practical issues such as organizational cohesion construction and identity conflict adaptation in university sports teams.

## 3 Method

### 3.1 Study design

From June to July 2025, a questionnaire survey and semi-structured interviews were conducted with university students from high-level sports teams across six universities in Nanjing, Jiangsu Province. This study adopted a mixed-method research design combining qualitative grounded theory analysis and quantitative fsQCA calculation Microsoft Corporation (2019).

This study adopted an exploratory sequential mixed-methods design. In the first qualitative phase, semi-structured interviews were undertaken to inductively identify context-specific core categories and multi-dimensional antecedent conditions of social identity coupling among high-level student-athletes. This grounded-theory step is essential, because the dual-role psychological mechanism within this special group is insufficiently theorized and may not be fully captured merely by pre-designed questionnaire scales. In the second quantitative phase, the core categories extracted from qualitative coding were operationalized as fuzzy-set variables for fsQCA analysis, which enables detection of equivalent configurational causal pathways that conventional net-effect statistical approaches may fail to identify.

The qualitative interview participants and the cases included in the formal fsQCA analysis are drawn from the same sampling pool. A subset of interview participants serves for formal qualitative coding and subsequent fsQCA modelling; additional interview participants are reserved solely for theoretical saturation testing and excluded from the fsQCA quantitative analysis. Detailed sample information, including participant numbers and demographic characteristics, is presented in Section 2.4 Research Sample.

In accordance with the Helsinki Declaration, this study was approved by the Institutional Ethics Review Committee of Nantong Institute of Technology (Approval No. NITI2025-PE028). All participants signed informed consent forms, and their personal privacy and legitimate rights were fully protected throughout the research process.

### 3.2 Research materials

#### 3.2.1 Grounded theory

As an inductive research paradigm, grounded theory breaks the limitations of hypothesis-driven confirmatory research (Sun et al., 2026; Glaser and Strauss, 1967), making it suitable for special groups such as high-level student-athletes who engage in both closed athletic training and open campus learning environments. It enables the bottom-up exploration of implicit psychological characteristics that are difficult to capture via traditional quantitative methods through in-depth analysis of first-hand empirical materials including semi-structured interviews, daily training observations, and campus life records (Yang and Shi, 2026).

This study adopts the core procedural path of “semi-structured anchoring—three-level coding refinement—theoretical model generation”(Bryant and Charmaz, 2007), following the main research thread of “dual-role attribute anchoring—classic framework embedding—mechanism logic extraction” to realize theoretical modeling based on real situational data of university students from high-level sports teams in China in Chinese universities.

#### 3.2.2 fsQCA

fsQCA adopts a configurational thinking paradigm that adapts to the complex nonlinear causal relationships of organizational psychology among university students from high-level sports teams in China, overcoming the limitations of traditional single-variable net effect analysis (Slaten et al., 2016).

This method takes multiple links of the “categorization-identification-comparison” framework as conditional variables, integrates the dual situational attributes of semi-formal athletic organizations and campus student groups, and identifies multiple equivalent influencing paths driving the organizational and educational psychological status of high-level student-athletes.

### 3.3 Social identity coupling model

Based on the literature review and theoretical framework elaborated above, this study constructs a localized social identity coupling model for university students from high-level sports teams in China.

The social identity coupling model for university students from high-level sports teams in China takes the classic “categorization-identification-comparison” framework of social identity theory as the core foundational framework, deeply anchoring the dual situational attributes of “semi-formal athletic organization + student group” to construct the underlying logic.

This study takes three mature standardized scales as the initial coding anchors: the 18-item perceived physical literacy instrument (PPLI, Ma et al., 2020), the 18-item Psychological Sense of School Membership (PSSM) scale (Sotardi and Rudolf, 2026), and the 9-item inclusive leadership questionnaire (ILQ, Cheng et al., 2022). Initial concepts are extracted from questionnaire feedback on physical literacy perception, sense of belonging, and inclusive leadership, and core categories covering identity categorization triggers, dual identity formation paths, and inter-group comparison behavioral characteristics are gradually refined through open, axial, and selective coding. Taking classical Social Identity Theory as the theoretical anchor, this study refines and extracts three potential triggering conditions for identity categorization in dual-role contexts: dual-dimensional perceived physical literacy kernel, dual-dimensional sense of belonging construction logic, and single-dimensional inclusive leadership openness attribute; two formation pathways of dual identification, namely the dual identity categorization conduction path and the inter-group comparison output path; as well as one behavioral feature of bidirectional intergroup comparison: the state of group-based social identity under dual-identity context.

Finally, fsQCA is applied to assign configurational values to core categories and identify multiple equivalent paths of positive social identity formation, constructing an integrated social identity coupling model with both empirical grounded attributes and nonlinear multi-configurational explanatory power for the interdisciplinary context of organizational and educational psychology. See the first part of Figure 1.

**Figure 1:**
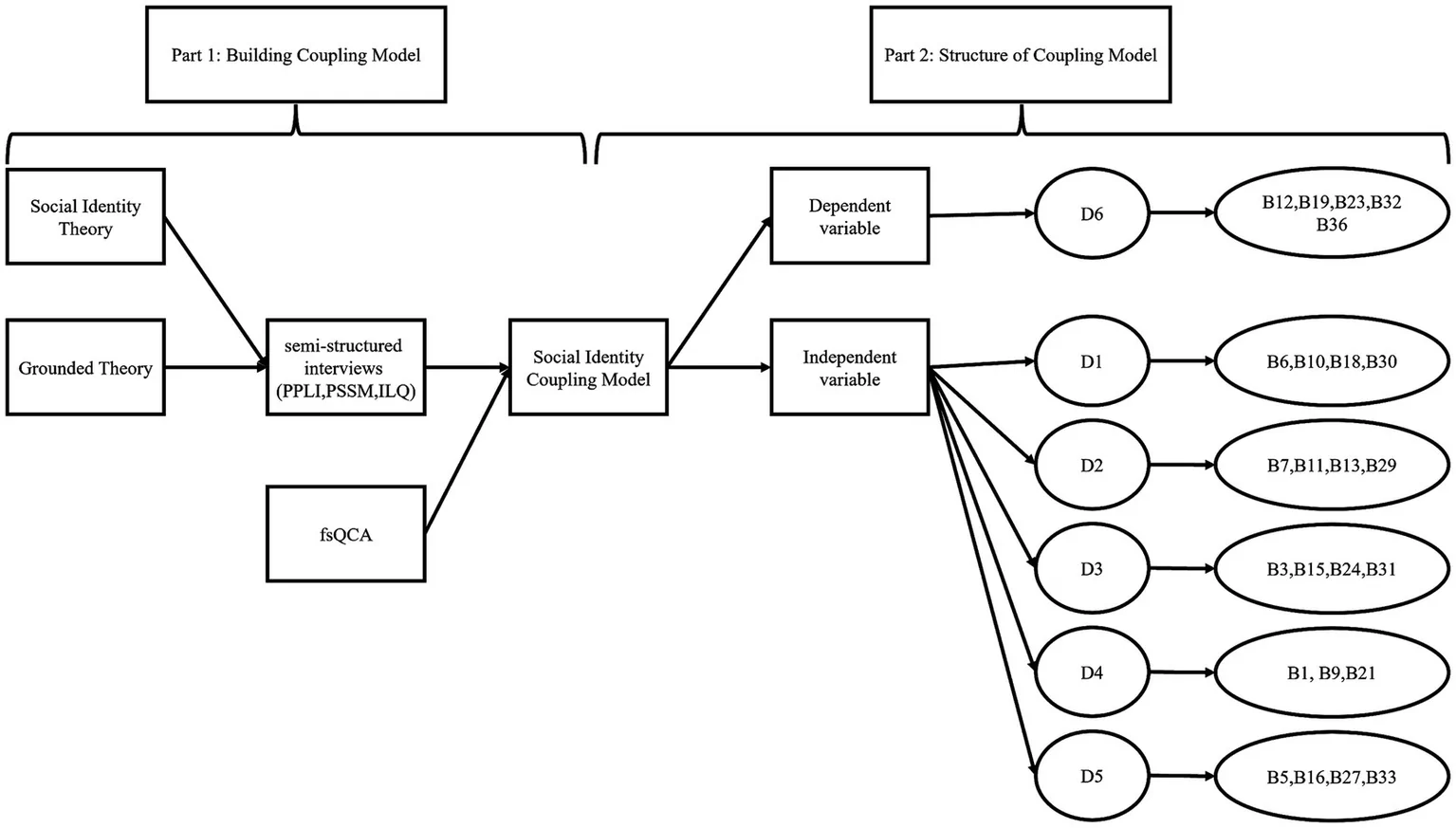
Social identity coupling model of university students from high-level sports teams in China. D1-D5, results of selective coding based on grounded theory, the five Level-1 independent variable of the coupling model, 24 Level-2 independent variables across five categories (axial coding B1–B33); D6, results of selective coding based on grounded theory; the dependent variables of the coupling model; five Level-2 dependent variables (axial coding B12–B36); fsQCA, fuzzy-set qualitative comparative analysis; PPLI, perceived physical literacy instrument; PSSM, Psychological Sense of School Membership; ILQ, inclusive leadership questionnaire.

### 3.4 Research sample

Adopting the maximum variation sampling strategy (Yang and Shi, 2026), this study initially recruited 76 university student-athletes from high-level sports teams across six universities, covering five sports categories including gymnastics, ball games, track and field, swimming, and other events. All participants were over 18 years old and possessed dual identities of first-class or above national sports athlete certification and full-time university students.

This sample size also meets the fsQCA small-sample adaptability criterion (10–40 cases with 3–5 cases per factor) (Slaten et al., 2016). This sample size meets multiple methodological requirements: it covers nine core factors derived from the three-dimensional frameworks of physical literacy, sense of belonging, and inclusive leadership, and ranges from 27 (27 = 9 × 3) to 45 (45 = 9 × 5), with an average valid sample size of 32 [32 ≈ (27 + 45) / 2]. Additionally, reserving 10% independent verification samples for grounded theory saturation testing (Yang and Shi, 2026), the final sample size was determined as 36 (36 = 32 + 4,4 ≈ 32*10%) participants. The sampling strategy and methods support the robust analysis of structural model antecedent conditions, ensure the consistency of qualitative and quantitative results, and satisfy the sampling coverage requirements.

Maximum-variation sampling was applied to build the target sample pool of 76 participants. Multiple stratification dimensions were adopted, including gender, age, university type, grade, academic major, degree, sport event, and athletic level, to maximize heterogeneity across cases. The theoretical number of cases per stratum was calculated as *n* = 36* N/76, where N denoted the number of cases within each stratum in the 76-person pool. Constrained by theoretical saturation for grounded theory and sample-size requirements for fs-QCA analysis, we prioritized representation across core dimensions (sport event and athletic level), which allowed minor departures from strict proportionality on demographic variables. Within each stratum, computer-generated random sequences were used for sampling without replacement, yielding the design/stratified recruitment sample of 36 participants.

In accordance with the theoretical saturation principle (12–20 participants) of grounded theory (Sun et al., 2026), 36 participants were randomly selected as the final research sample from the design/stratified recruitment sample.

Demographic information including gender, age, university, grade, academic major, degree, sport event, and athletic level was collected for each participant to verify sample representativeness and ensure research rigor. Table 1 presents the seven demographic variables of the 36 participants using percentages to clarify sample representativeness. Demographic variables were not incorporated into the model factor system; their statistical analysis served only for sample accuracy screening and research rigor demonstration.

**Table 1:** Demographic characteristics of university students from high-level sports teams in China.

Item	Target sample – *N*	Target sample – %	Random sample – *N*	Random sample – %
Gender				
Female	35	46.05%	24	66.67%
Male	41	53.95%	12	33.33%
Age				
18-19 years old	32	42.11%	10	27.78%
20-21 years old	16	21.05%	12	33.33%
22 years old and above	28	36.84%	14	38.89%
University				
Project 985 University	15	19.74%	5	13.89%
Project 211 University	22	28.95%	11	30.56%
Double First-Class University	26	34.21%	13	36.11%
Comprehensive University	13	17.11%	7	19.44%
Grade				
Grade 1	28	36.84%	13	36.11%
Grade 2	30	39.47%	15	41.67%
Grade 3	11	14.47%	6	16.67%
Grade 4	7	9.21%	2	5.56%
Academic major				
Science	24	31.58%	8	22.22%
Engineering	21	27.63%	11	30.56%
Arts and Humanities	31	40.79%	17	47.22%
Degree				
Bachelor	42	55.26%	22	61.11%
Master	31	40.79%	12	33.33%
Doctor	3	3.95%	2	5.56%
Sport event				
Gymnastics	25	32.89%	6	16.67%
Ball Games	32	42.11%	16	44.44%
Athletics	8	10.53%	7	19.44%
Swimming	6	7.89%	5	13.89%
Other Sports	5	6.58%	2	5.56%
Athletic level				
National level 1 athlete	46	60.53%	21	58.33%
National Master of Sports	28	36.84%	14	38.89%
International Master of Sports	2	2.63%	1	2.78%

### 3.5 Data collection and organization

#### 3.5.1 Data collection

A flexible semi-structured interview outline was designed around the core research questions of the social identity coupling model (see Table 2A). The interview scope covered nine core dimensions derived from the three antecedent categories of physical literacy (PPLI), sense of belonging (PSSM), and inclusive leadership (ILQ), which were established as the variable boundaries of the social identity model. It is important to note that the nine core dimensions derived from the three prerequisite categories were neither added to nor removed; all were retained. No fixed closed answers were set, allowing adjustable questioning sequences and wording to adapt to on-site interaction. The outline was revised through two rounds of pre-interviews with three experienced participants to ensure scientific rigor. Individual in-depth interviews were conducted in private appointed settings with full audio recording and auxiliary written records. A dual-researcher protocol was adopted: one researcher led the interview while the other took field notes on key information. The note-taker did not intervene in the interview process unless clarification was needed, thereby preserving the natural flow of the conversation.

**Table 2:** Statistical table of data based on grounded theory.

(A) Semi-structured interview for the social identity coupling model	
Dimensions	Description
I. Physical literacy (Variable 1)	
Dimension 1: Motivation and confidence	1. When participating in collective or group-based physical activities, what is your willingness to get involved? What factors facilitate or constrain your motivation to participate?
	2. For group tasks that demand physical engagement, how confident are you about your performance? How does this confidence level influence your participation and behavior within the group?
Dimension 2: Physical competence	1. To what extent do your basic physical fitness, motor skills and physical adaptability meet the practical requirements of routine group activities and collective tasks? Please illustrate with specific examples.
	2. How do your strengths or limitations in physical competence affect your group integration and teamwork when activities involve physical demands?
Dimension 3: Knowledge and understanding	1. To what extent do you possess knowledge about scientific exercise, physical conditioning and health protection? How does such knowledge guide your engagement in group-based physical activities?
	2. How does your awareness of your physical status, physical patterns and physical limitations shape your self-positioning, participation choices, and subjective experience of social identity within the group? Please elaborate.
II. Sense of belonging (Variable 2)	
Dimension 4: Social identity	1. To what extent do you recognize and accept your membership in this group? How does this group-member identity shape your behaviors and attitudes within the collective?
	2. Compared with external groups, do you develop a distinct sense of group identity for your in-group? What behaviors reflect this group-based social identity?
Dimension 5: Belonging	1. During daily interactions and group activities, to what extent do you feel accepted and included by the group? How does this perceived acceptance reinforce your sense of belonging?
	2. Do you feel genuinely integrated and sustain a stable sense of belonging in this group? Have you experienced marginalization or a lack of belonging?
Dimension 6: Attachment	1. Over time, have you developed emotional attachment toward the group and its members? In what situations or observable ways is this attachment manifested?
	2. If you were to leave or switch groups, would you experience feelings of reluctance? How does this group attachment influence your social identity and willingness to participate?
III. Inclusive leadership (Variable 3)	
Dimension 7: Openness	1. In routine group operations and activities, to what extent do leaders maintain an open-minded stance toward diverse viewpoints, new ideas and dissenting opinions from members? Please provide concrete examples.
	2. When members propose suggestions inconsistent with existing group decisions or leaders’ preferences, how do leaders typically respond? To what extent are divergent perspectives respected and incorporated? How does such openness affect your sense of engagement?
Dimension 8: Availability	1. When you encounter difficulties or confusion in group-related affairs, are leaders able to offer timely resources, assistance and guidance? How sufficient and relevant is the support provided?
	2. To what extent do leaders’ support and available resources match members’ practical needs? How do these available supports influence your recognition and sense of belonging toward the group?
Dimension 9: Accessibility	1. How accessible and approachable do you perceive your leaders to be? Can you comfortably and efficiently raise concerns, express needs and communicate ideas with them?
	2. How does leader accessibility influence your participation motivation, emotional investment and group-based social identity? What subjective differences do you perceive between approachable versus distant leadership styles?
IV. Supplementary open-ended questions	
	1. Based on your experience, to what extent do physical literacy, sense of belonging and inclusive leadership interact with one another? Please describe these inter-relationships.
	2. Beyond the themes discussed above, what additional factors may shape your social identity within the group?
	3. What adjustments to group climate, leadership practices or individual conditions could enhance members’ social identity and sense of belonging?

(B) Open coding	
Code	Label
A1	Perception of Sports Team Identity Label
A2	Frequency of Daily Training Participation
A3	Perception of Coaches’ Inclusive Behaviors
A4	Difficulty in Integrating into Campus Courses
A5	Frequency of Teammate Interaction
------	------
A169	Experience of Humanistic Care from Coaches’ Proactive Attention to Athletes’ Daily Emotional State
A170	Perception of Identity Transition Support from School-arranged Pairing Assistance During the Retirement Transition Period
A171	Public Affairs Participation Behavior in Proactively Participating in the Management of Sports Team Student Associations
A172	Perception of the Rules of the Dual Graduation Criteria for Training and Academics Implemented by Sports Teams
Examples	
Interview	II. Sense of Belonging (Independent Variable 2) 1. During daily interactions and group activities, to what extent do you feel accepted and included by the group? How does this perceived acceptance reinforce your sense of belonging?
Answer	1. As elite student-athletes on the university team, we are exempted from some general compulsory courses and are not required to earn full credits for these courses like ordinary students, which represents a preferential policy exclusively offered to student-athletes by the university. 2. I can clearly perceive this special treatment, which alleviates academic pressure caused by time conflicts with training and competitions and allows us to devote more time to specialized athletic training.
Code	1. Coding anchoring: The two sentences consist of three components: (1) subjects: high-level student-athletes; (2) objective institutional policy: exemption from selected general compulsory courses; (3) core construct: individuals’ subjective perceptions of preferential institutional arrangements. 2. Text interpretation: The first quote presents factual institutional context and contrasts credit requirements between student-athletes and regular undergraduates. The second quote captures subjective experience, illustrating how the policy mitigates academic-training conflicts and operationalizes the core “perception” element of this code. 3. Open-coding node: A105 Perceptions of special treatment granted to athletes in the form of exemptions from certain general education courses. 4. Fit justification: The two excerpts establish a logical sequence from institutional reality to subjective perception, fully capturing the meaning scope of code A105. The material is suitable as raw quotation for open coding in grounded-theory analysis.

(C) Axial coding	
Code	Label
B1	Perception of In-group Identity Categorization
B2	Characteristics of Training Behavior Engagement
B3	Perception of Organizational Inclusive Behaviors
B4	Characteristics of Campus Academic Adaptation
B5	Quality of In-group Interaction
------	------
B34	Perception of Barriers to Physical Literacy Development
B35	Experience of Lack of Inclusive Climate
B36	Perception of Dual-Identity Synergy

(D) Selective coding and model dimensions		
Selective code	Model dimensions	Label
C1	D1	Dual-dimensional perceived physical literacy kernel
	B6	Self-perception of physical literacy
	B10	Characteristics of daily exercise behavior
	B18	Self-assessment of motor skills
	B30	Autonomous exercise planning ability
C2	D2	Dual-dimensional sense of belonging construction logic
	B7	Dual identity conflict experience
	B11	Dual identity belonging experience
	B13	Perception of athletic identity
	B29	Perception of student identity
C3	D3	Single-dimensional inclusive leadership openness attribute
	B3	Perception of organizational inclusive behavior
	B15	Perception of differentiated support
	B24	Perception of evaluation fairness
	B31	Positive perception of inclusive climate
C4	D4	Dual identity categorization conduction path
	B1	Perception of in-group identity categorization
	B9	Degree of organizational rule cognition
	B21	Degree of in-group identity consensus
C5	D5	Inter-group comparison output path
	B5	Quality of in-group interaction
	B16	Willingness for cross-group interaction
	B27	Positive experience of intergroup comparison
	B33	Negative experience of intergroup comparison
C6	D6	State of group-based social identity under dual-identity context
	B12	Perception of group-based academics-training conflict
	B19	Negative experience of group-based identity rejection
	B23	Experience of group-based belonging-deficit
	B32	Characteristics of group-based campus social integration
	B36	Perception of group-based dual-identity synergy

Strict ethical guidelines were implemented: all participants signed informed consent forms with clear notification of research purposes, information usage scope, and unconditional withdrawal rights. All personal sensitive information was strictly confidential, and inductive questions causing psychological discomfort were avoided throughout the process.

A dual-person quality control mode was adopted: one researcher dominated the interview while another recorded key information. All audio records were transcribed verbatim within 24 h. All transcripts were cross-checked by a third researcher, with participant feedback triangulation verification to guarantee data accuracy and validity. Each participant was assigned a unique coded number (1–36) for supplementary re-interview if needed.

#### 3.5.2 Data organization

Following the sequential analytical logic of prior mixed-methods studies integrating grounded theory and fsQCA, this section focuses on the complete data flow and cross-stage linkage operations from qualitative coding to quantitative analysis. The methodological foundations and calculation rules for each stage will be elaborated in the Data Calculation section and are not repeated here. This section only clarifies the input–output relationships, format conversion rules, and parallel processing workflows across stages, ensuring traceability and consistency between qualitative coding results and fsQCA quantitative calculations.

##### 3.5.2.1 Data flow overview

The data processing in this study follows a linear progressive workflow of “qualitative coding → variable assignment → data calibration → configurational analysis → robustness verification.” The input–output relationships across stages are as follows: (1) The 36 verbatim transcripts produced in the data collection stage undergo three-level coding, yielding concepts, main categories, and core categories identified by the A/B/C labeling system; (2) Coding results are mapped into nine Level-1 independent variables and one dependent variable according to the variable definition rules; (3) Following the assignment rules, variable assignment is completed through Excel database queries, generating the original dataset (Dataset-A) and the mean-imputation dataset (Dataset-M); (4) Following the direct calibration method, assigned data are converted into [0,1] fuzzy-set membership scores; (5) Calibrated data are imported into fsQCA 4.1 to perform necessary condition analysis and configurational path analysis; (6) fsQCA output results are imported into R 5.3 to perform ceiling regression (CR) and ceiling envelopment (CE) analyses; (7) Finally, dual-dataset robustness testing is completed. In this workflow, the structured output of each stage serves as the input for the subsequent stage, forming a closed-loop data chain.

##### 3.5.2.2 Mapping from coding labels to fsQCA variables

Data coding: This study adopted the classic three-level coding procedure (open coding, axial coding, and selective coding) of grounded theory. The “categorization-identification-comparison” framework of social identity theory served as the fundamental logical anchor throughout the coding process, adapting to the dual situational attributes of university students from high-level sports teams in China. Three standardized scales were embedded as coding anchors to ensure dimensional consistency between qualitative coding and subsequent quantitative analysis. Unified coding labels were adopted: uppercase English letters (A, B, C) plus Arabic numerals and conceptual descriptions, where numerals represented serial numbers of open coding conceptual description, axial coding main categories, and selective coding core categories, respectively.

Reliability and validity verification: Theoretical saturation testing was implemented for data cleaning to verify the integrity of theoretical constructs and data reliability (Yang and Shi, 2026). The 36 samples were randomly divided into 32 formal coding samples (90%) and 4 independent saturation test samples (10%) (Yang and Shi, 2026). Methodologically, the principle of theoretical saturation holds that data reach saturation when the post-hoc split-sample fail to generate novel concepts, categories, or relationships among categories (Yang and Shi, 2026). A standardized three-round independent coding-review-verification procedure was conducted by three researchers to eliminate subjective coding bias. This procedure ensured the validity and reliability of qualitative results.

A strict hierarchical mapping relationship exists between the label system produced by three-level coding and fsQCA variables. Selective coding core categories (C-series labels) directly correspond to the Level-1 independent variables of the model, comprising nine antecedent conditions belonging to three antecedent categories: physical literacy (perceived motor competence, motivation and confidence, knowledge and understanding), sense of belonging (emotional bonding, team acceptance, value identification), and inclusive leadership (openness, effectiveness, leader recognition). The social identity coupling level, as the dependent variable, derives from the core category of “group-based social identity state” in selective coding. Axial coding main categories (B-series labels) correspond to the Level-2 sub-dimensions under each Level-1 independent variable. The number of B categories (Fu et al., 2026; Diao et al., 2024; Yan et al., 2022) under each core category determines whether the variable adopts a 4-point, 5-point, or 6-point Likert assignment standard. Open coding conceptual descriptions (A-series labels) provide the original counting basis for frequency statistics of B categories; the occurrence frequency of multiple A-labels under the same B-category is counted toward the coefficient of that sub-dimension. This mapping ensures traceability from qualitative concepts to quantitative variables, as every fsQCA variable can be traced back to a specific set of coding labels.

##### 3.5.2.3 Excel assignment database construction and querying

To objectify and ensure reproducibility of assignment calculations, this study constructed a structured assignment database in Excel 2019, comprising three worksheets: SHEET1 (assignment rule table), SHEET2 (coding count table), and SHEET3 (assignment result table). SHEET1 stores the continuous variable assignment rules, with fields including: variable name, number of sub-dimensions, corresponding Likert scale points, and base values for each presence level (from 0 to all sub-dimensions). SHEET2 stores the axial coding counts of 32 formal coding samples under each variable, with fields including: sample ID (P01–P32), variable name, B-category label, and occurrence frequency. SHEET3 automatically completes assignment calculations through the IFERROR [VLOOKUP ()] function: first, using “variable name + number of present sub-dimensions” as a composite query key to match the corresponding base value from SHEET1; then, using the sum of occurrence frequencies of B-categories under that variable in SHEET2 as the coefficient; finally, outputting the assignment result of “base × coefficient.” If a query key has no match (e.g., missing coding), IFERROR returns a blank value and marks it for imputation. This database query method avoids subjective errors in manual calculation, and all assignment results can be traced back to original coding counts through formulas.

##### 3.5.2.4 Dual-dataset construction and parallel analysis workflow

Based on the data imputation strategy, this study constructed two parallel datasets to systematically evaluate the impact of zero-value treatment on results. Dataset-A (original dataset) retains the original zero values from coding assignment, treating zero-coded entries as genuine observations. Dataset-M (imputation dataset) replaces all zero-coded values with the variable-specific sample mean computed across 32 valid samples. The construction steps for both datasets are: Step 1, based on the SHEET3 assignment results, duplicate and generate two identical initial data matrices; Step 2, for Dataset-M, compute the sample mean of non-zero values column by column, use the Excel IF function to replace zero values with the corresponding column mean, while Dataset-A remains unchanged; Step 3, perform Pearson correlation sensitivity analysis on both datasets, testing the criteria of |r| > 0.7 and *p* < 0.001 to confirm the degree of overall sample structure preservation (Tajfel and Turner, 1979); Step 4, both datasets independently execute the full fsQCA procedure with identical calibration parameters (0.95/0.50/0.05) and analytical parameters (consistency threshold 0.80, case frequency 2); Step 5, use Dataset-M as the primary analysis dataset for reporting main results, and Dataset-A as an alternative measurement specification for cross-dataset robustness comparison. The dual-dataset parallel design ensures that research conclusions do not depend on a single missing-value treatment strategy.

#### 3.5.3 Quantitative calculation of social identity coupling model

fsQCA 4.1 and R 5.3 software were adopted for quantitative calculation and empirical testing of the social identity coupling model (Pappas and Woodside, 2021).

The quantitative analysis included four standardized steps: first, variable definition, assignment, and standardization; second, necessary condition (single necessary condition verification, non-necessary condition verification and bottleneck level testing); Third configurational path analysis; fourth, the robustness testing.

The following describes the details of the calculation:

##### 3.5.3.1 Variable definition

In the fsQCA calculation, the social identity of university students from high-level sports teams in China was first included as the dependent variable of the model, and the other core categories of selective coding results were taken as the Level-1 independent variables. Furthermore, the axial encodings under its selective encoding are chosen as Level-2 independent variables in the coupling model.

##### 3.5.3.2 Variable assignment

For quantitative calculations of multidimensional models, based on the methodological framework of multidimensional conceptual measurement, the standard for continuous variables is incorporated as the base, and the frequencies of the sub-variables are further incorporated as coefficients. The product of the base and the coefficient is used as the numerical value of the variable to complete the variable assignment (Yang and Shi, 2026). Continuous variable assignment criteria matched 4–6-point Likert scale levels according to the sub-dimensional quantity of each core category (Yang and Shi, 2026). A 4-point scale was used for three sub-dimensions, a 5-point scale for four sub-dimensions, and a 6-point scale for five sub-dimensions (Yang and Shi, 2026).

When a variable dimension (selective coding core categories) has three sub-dimensions (axial coding main categories), a 4-point Likert scale is adopted for variable assignment: 0 (no axial coding main categories present), 0.33 (one axial coding main categories present), 0.67 (two axial coding main categories present), and 1 (all three axial coding main categories present).

When a variable dimension (selective coding core categories) has four sub-dimensions (axial coding main categories), a 5-point Likert scale is adopted for variable assignment: 0 (no axial coding main categories present), 0.25 (one axial coding main categories present), 0.50 (two axial coding main categories present), 0.75 (three axial coding main categories present), and 1 (all four axial coding main categories present).

When a variable dimension (selective coding core categories) has five sub-dimensions (axial coding main categories), a 6-point Likert scale is adopted for variable assignment: 0 (no axial coding main categories present), 0.20 (one axial coding main categories present), 0.40 (two axial coding main categories present), 0.60 (three axial coding main categories present), 0.80 (four axial coding main categories present), and 1 (all five axial coding main categories present).

This clearly explains the reasons for including variable assignments in the base number: First, there may be instances of multiple occurrences of the same sub-dimension within a variable; second, these multiple occurrences represent a “one-to-many” phenomenon that takes into account both axial coding and open coding in grounded theory; and third, cardinality addresses the shortcomings of single assignments (the continuous variable method). For example: If a respondent has three axial coding (including one B1 and two B2) under a particular selective coding (such as D2, which is based on a 5-point Likert scale), then the base number for his or her D2 variable is 0.75, the coefficient is 3, and the value is 2.25 (2.25 = 0.75 × 3); The coefficient of 3 represents not only the results of the three axial coding but also the structure of those three axial coding, as well as the results and structure of the three open coding.

##### 3.5.3.3 Data calibration

Variable data calibration was standardized using the direct calibration method with completely dependent (0.95), intersection point (0.50), and completely dependent (0.05) thresholds (Pappas and Woodside, 2021).

##### 3.5.3.4 Necessary condition

The calculation of necessary conditions for the model is divided into two steps: single necessary condition verification, non-necessary condition verification and bottleneck level testing of configurational paths.

Single necessary condition verification: It was conducted after data standardization, with an inclusion criterion of fsQCA independent variable results for high/low social identity greater than 0.9 (Pappas and Woodside, 2021).

Non-necessary condition verification: Both ceiling regression (CR) and ceiling envelopment (CE) analysis based on R language were adopted for non-necessary condition verification, with criteria of effect size d > 0.1 and *p*-value *p* < 0.01 (Fu et al., 2026; Dul, 2015).

Bottleneck level testing: The bottleneck level testing of the model is to evaluate the sensitivity characteristics of the social identity coupling model for university students from high-level sports teams in China. A five-level gradient sensitivity analysis was conducted to identify the minimum threshold constraints of each antecedent variable under different social identity levels (Tajfel and Turner, 1979). The bottleneck level test results demonstrated the sensitivity characteristics of the social identity coupling model for university students from high-level sports teams in China, providing data foundation and quantitative basis for the qualitative evaluation of the practical value and public service social value of this study.

##### 3.5.3.5 Configurational paths

The configurational paths of the model are nonlinear combinations of multiple independent variables. Three types of fsQCA solutions were generated: complex solution, parsimonious solution, and intermediate solution (Pappas and Woodside, 2021). Following established standards, intermediate solutions were adopted as the dominant configurational paths, and parsimonious solutions were used to identify core and peripheral conditions. A condition was identified as core when it appeared in both parsimonious and intermediate solutions; otherwise, it was classified as peripheral. Conditions appearing only in intermediate solutions were classified as peripheral conditions with presence/absence status. Strict truth table thresholds were set: consistency >0.75, overall coverage >0.5, and overall consistency >0.75 (Pappas and Woodside, 2021; Schneider and Wagemann, 2012).

##### 3.5.3.6 Robustness testing

To examine the sensitivity of the findings to missing-value treatment, we constructed two parallel datasets. Dataset-A retained the original questionnaire coding, treating zero-coded entries (unanswered or non-applicable items) as genuine observations. Dataset-M replaced all zero-coded values with the variable-specific sample mean computed across valid responses, thereby eliminating potential systematic bias introduced by missing-value markers in set-membership calibration. Both datasets contained identical cases (*N* = 32), antecedent conditions, and outcome variables, differing only in the treatment of zero-coded entries. We used Dataset-M as the primary analysis dataset because it adopts a more conservative measurement approach that reduces the distorting effect of extreme zero values on fuzzy-set calibration; Dataset-A served as an alternative measurement specification for cross-dataset robustness testing.

Primary analysis dataset (Dataset-M) robustness test: Robustness testing was realized by raising the consistency threshold from 0.80 to 0.85 and adjusting the case frequency threshold from 2 to 3 (Tajfel and Turner, 1979). Evaluation indicators included overall coverage > 0.5, overall consistency > 0.75, and stable core configurational structure (Tajfel and Turner, 1979).

Cross-dataset (Dataset-A) robustness test: Configurational-level outcomes: we independently executed the full fsQCA procedure—including separate calibration, Single necessary condition analysis, and sufficiency configurational path analysis—for each dataset and compared whether core conditions, configuration structures, overall solution consistency, overall coverage and raw coverage remained stable. In the fsQCA analyses, both datasets employed identical analytical parameters and threshold settings, including data calibration, analysis of individual necessary conditions, truth table construction, and configuration path extraction. All parameters were held constant across the two datasets to ensure that any differences in configurational outcomes originated solely from the zero-coding treatment rather than from changes in analytical parameters.

## 4 Results

### 4.1 Data coding results

#### 4.1.1 Open coding

A total of 172 initial open coding concepts (A1–A172) were extracted, covering multiple dimensions including identity cognition, study-training conflict, coach and teacher support, intra-team interpersonal interaction, cross-group campus socialization, physical health behavior, and institutional perception (see Table 2B).

The example section in Table 2B illustrates the detailed process of open coding, including a semi-structured interview and the process of transcribing the interviewee’s remarks, as well as four coding practices. The core node for this open coding session is A105: Perception of Special Treatment Granted to Athletes Through Exemption from Certain General Education Courses. The two textual excerpts form a complete logical loop of objective policy facts - subjective individual perceptions, fully capturing the entire core connotation of Code A105. They not only reflect the exclusive institutional privileges attached to student-athlete identity, but also demonstrate individuals’ genuine cognition and experience of such preferential treatment. Consistent with the text-matching criteria for open coding in qualitative research, these excerpts can be directly employed for subsequent grounded-theory coding analysis and dimension extraction.

#### 4.1.2 Axial coding

Axial coding was conducted on 172 initial concepts, yielding 36 core axial categories (B1–B36) that systematically integrated scattered initial codes and covered all core research dimensions of student-athlete psychology and situational experience (see Table 2C). A105 (Perceptions of special treatment granted to athletes through exemptions from certain general education courses)-B27 (Positive experience from intergroup comparison), as an example of the axial encoding process: According to the classic categorization-identification-comparison framework, institutional policy (exemption from general compulsory courses) first facilitates individuals’ social categorization. Subsequently, individuals develop in-group identification as student-athletes. Through intergroup comparison, positive intergroup experiences are ultimately generated. Perceptions of special treatment (Code A105) serves as a critical psychological mediator linking objective institutional arrangements to the positive experiences of Code B27, which realizes the logical connection between the main category and sub-category in axial coding. The 36 axial coding categories systematically integrated scattered initial codes, comprehensively covering core research dimensions such as identity cognition, study-training relationship, campus support, interpersonal interaction, self-development, and inter-group experience of university students from high-level sports teams in China.

#### 4.1.3 Selective coding

Six core main categories (C1–C6) were finally extracted via selective coding, constituting the six-dimensional core framework of the social identity model (see Table 2D). The core categories included dual-dimensional perceived physical literacy kernel (C1), dual-dimensional belongingness construction logic (C2), single-dimensional inclusive leadership openness attribute (C3), dual identity categorization conduction path (C4), inter-group comparison output path (C5) and state of group-based Social Identity under Dual-identity Context (C6), fully covering 24 axial coding connotations. B27 (positive experience from intergroup comparison)-C5 (inter-group comparison output path), as an example of the axial coding process: Selective coding develops the core storyline, in which B27 (positive experience from intergroup comparison) acts as the pivotal category, and C5 (inter-group comparison output path) outcomes serves as the outcome transmission pathway under the core category. This analysis focuses on the formation pathway of dual identity (student-athlete identity identification + ordinary undergraduate identity identification) among high-level student-athletes, and illustrates how positive experiences generated by intergroup comparison shape the construction of dual identity via the C5 output pathways.

#### 4.1.4 Reliability and validity verification

The 36 semi-structured interview samples were divided into 32 formal coding samples (90%) and 4 independent random saturation test samples (10%, Yang and Shi, 2026), and open, axial, and selective coding were completed using grounded theory. Adopting a post-hoc split-sample saturation procedure, this study conducted segment-by-segment coding for the four reserved hold-out samples and cross-referenced against the established categorical framework after completing three-level coding for primary samples. Axial and selective coding were not repeated for hold-out samples. No new concepts, categories or inter-category connections emerged from the reserved samples. Accordingly, theoretical saturation was achieved in the present study. Sensitivity assessment based on multiple lines of evidence reveals that the overall pattern of statistical significance remains largely consistent between Dataset-A and Dataset-M. Therefore, findings derived from Dataset-M can be used to the social-identity coupling model characteristics of dual identities among university students from high-level sports teams in China.

### 4.2 Social identity coupling model empirical results

#### 4.2.1 Variable definition

The five core categories [D1 (dual-dimensional perceived physical literacy kernel) to D5 (inter-group comparison output path], independent variables) form a logically hierarchical and mutually complementary structural system, with D6 (state of group-based social identity under dual-identity context) as the dependent variable, fully interpreting the internal mechanism, influence path, and outcome characteristics of dual-role development among high-level student-athletes. See the second part of Figure 1.

The independent variable system of the integrated model in this study follows the standard hierarchical logic of grounded theory. Five first-order core categories extracted through selective coding serve as the top-level independent variables, each encompassing second-order observational subcategories clustered through axial coding, thereby forming a standard nested variable system of “selective coding high-order core—axial coding low-order dimension.” The specific hierarchical correspondences are as follows:

D1 (Dual-dimensional Perceived Physical Literacy Kernel), as the outcome of the first selective coding and the first core independent variable (C1), indicates the comprehensive perceived physical literacy kernel formed by individuals across multiple dimensions of self-cognition, behavioral performance, skill mastery, and autonomous planning. It serves as the foundational core variable for exercise behavior and physical self-development. It comprises four second-order observational dimensions (sub-independent variables), namely the following four axial coding subcategories: B6 (Self-perception of Physical Literacy), B10 (Characteristics of Daily Exercise Behavior), B18 (Self-assessment of Motor Skills), and B30 (Autonomous Exercise Planning Ability).

D2 (Dual-dimensional Sense of Belonging Construction Logic), as the outcome of the second selective coding and the second core independent variable (C2), indicates the bidirectional construction process and internal logic of sports group belonging, whereby individuals, within the dual-role context of student identity and athletic identity, accomplish this construction through identity conflict adjustment, group belonging perception, athletic identity, and identity categorization. It comprises four second-order observational dimensions (sub-independent variables), namely the following four axial coding subcategories: B7 (Dual Identity Conflict Experience), B11 (Dual identity belonging experience), B13 (Perception of athletic identity), and B29 (Perception of student identity).

D3 (Single-dimensional Inclusive Leadership Openness Attribute), as the outcome of the third selective coding and the third core independent variable (C3), indicates individuals’ overall perception of inclusive management and leadership behaviors in sports organizational contexts. It encompasses four openness traits—organizational inclusive behavior, differentiated support, evaluation fairness, and inclusive climate—and serves as the core representational variable of the organizational inclusive environment. It comprises four second-order observational dimensions (sub-independent variables), namely the following four axial coding subcategories: B3 (Perception of Organizational Inclusive Behavior), B15 (Perception of Differentiated Support), B24 (Perception of Evaluation Fairness), and B31 (Positive Perception of Inclusive Climate).

D4 (Dual Identity Categorization Conduction Path), as the outcome of the fourth selective coding and the fourth core independent variable (C4), indicates the internal path mechanism through which individuals, based on group identity categorization cognition, organizational rule cognition, and in-group identity consensus, achieve the internal conduction, integration, and consolidation of dual identity cognition. It comprises three second-order observational dimensions (sub-independent variables), namely the following three axial coding subcategories: B1 (Perception of In-Group Identity Categorization), B9 (Degree of Organizational Rule Cognition), and B21 (Degree of In-group Identity Consensus).

D5 (Inter-group Comparison Output Path), as the outcome of the fifth selective coding (C5) and the fifth core independent variable, indicates the intergroup comparison output mechanism at the attitudinal, cognitive, and behavioral levels, whereby individuals generate positive and negative intergroup comparison experiences through in-group interaction and cross-group interaction behaviors. It comprises four second-order observational dimensions (sub-independent variables), namely the following four axial coding subcategories: B5 (Quality of In-group Interaction), B16 (Willingness for Cross-group Interaction), B27 (Positive Experience of Intergroup Comparison), and B33 (Negative Experience of Intergroup Comparison).

D6 (state of group-based social identity under dual-identity context) serves as the outcome of the sixth selective coding (C6) and the outcome variable of the coupled model. It describes the comprehensive psychological state of group-based identification formed among university students from high-level sports teams in China under the intertwined athlete-student dual-identity context. This outcome is represented by five axial-coding sub-dimensions: B12 (Perception of group-based academics-training conflict), B19 (Negative experience of group-based identity rejection), B23 (Experience of belonging-deficit), B32 (Characteristics of group-based campus social integration), and B36 (Perception of group-based dual-identity synergy). Among them, perception of academics-training conflict and perception of dual-identity synergy reflect the tension and compatibility between multiple social identities; negative experience of identity rejection and experience of belonging-deficit capture affective-evaluative responses stemming from group-related treatment; characteristics of campus social integration indicate individuals’ interactive involvement within group settings. Importantly, outcome D6 (state of group-based social identity under dual-identity context) is conceptually distinct from antecedent D2 (Dual-dimensional sense of belonging construction logic). D2 (Dual-dimensional sense of belonging construction logic) refers to the formative mechanisms and pathways through which the sense of belonging emerges and operates, acting as a process-oriented antecedent for configurational analysis. By contrast, D6 (state of group-based social identity under dual-identity context) represents the end-state of identification shaped jointly by multi-identity experiences and group interactions, functioning as the explained outcome in fsQCA.

The core categories exhibit clear logical hierarchies and complementary interrelationships, enabling systematic interpretation of the internal mechanisms, influence paths, and outcome characteristics of dual identity development among university students from high-level sports teams in China. Furthermore, the model has passed theoretical saturation testing, demonstrating good scientific rigor and integrity.

Accordingly, the fsQCA calculation comprises one outcome variable (D6 Social identity), 5 sub-dependent variables [B12 (Perception of academics-training conflict)-B36 (Perception of dual-identity synergy)], five conditional conditions [D1 (Dual-dimensional perceived physical literacy kernel) - D5 (Inter-group comparison output path)], and 19 sub-independent variables [B1 (Perception of in-group identity categorization) - B33 (Negative experience of intergroup comparison)].

#### 4.2.2 Variable assignment

Variable assignment: Based on the differences in the number of sub-dimensions across core dimensions, this study matched 4-point, 5-point, and 6-point Likert scale standards, respectively, (Yang and Shi, 2026), and conducted assignment for the five core independent variables from D1 (dual-dimensional perceived physical literacy kernel) to D5 (inter-group comparison output path). Combined with the coding emergence frequency of 32 main samples, initial variable assignment was completed for all cases, with original values ranging from 0 to 1, precisely corresponding to the actual presentation of core categories in each case. The weighted total score of the dependent variable ranged from 0 to 4.2. After standardized data imputation, the final valid data interval for each independent variable was 0.066–1.5, and the total score of the social identity dependent variable ranged from 0.2 to 4.2, with significant data gradient differences that effectively improved sample discrimination.

Sensitivity analysis: Pearson correlations were computed across Dataset-A (retaining zeros) and Dataset-M (zero-values imputed with means) (see Table 3A). Case-level correlations for each condition across two datasets ranged from 0.944 to 0.993 (all *p* < 0.001), indicating limited perturbation to individual-case rank-order after re-assignment. Comparing inter-variable correlation patterns between the two datasets, most pairwise associations remained stable. The notable change occurred between D2 (Dual-dimensional sense of belonging construction logic) and D6 (State of group-based social identity under dual-identity context): their significant moderate correlation in Dataset-A (*r* = 0.484, *p* = 0.005) became non-significant in Dataset-M (*r* = 0.292, *p* = 0.105). This suggests raw zero observations partly inflated their empirical association, consistent with the theoretical concern about conceptual overlap between the two constructs. No substantial shifts were observed for other antecedent conditions. Given numerical overflow prevented complete fuzzy-set calibration for Dataset-A, sensitivity evidence further included multi-anchor robustness checks within Dataset-M and qualitative reflection on the substantive meaning of zero-coded entries from grounded-theory coding.

**Table 3:** FsQCA of the social recognition model for university students from high-level sports teams in China.

(A) Sensitivity analysis of Pearson correlations across Dataset-A (retaining zeros) and Dataset-M (zero-values imputed with means)			
Cross-validation	*r*	*p*	Interpretation
DA6 ↔ DM6	0.944	*p* < 0.001	Extremely high
DA1 ↔ DM1	0.973	p < 0.001	Extremely high
DA2 ↔ DM2	0.993	p < 0.001	Almost identical
DA3 ↔ DM3	0.973	p < 0.001	Extremely high
DA4 ↔ DM4	0.977	p < 0.001	Extremely high
DA5 ↔ DM5	0.984	p < 0.001	Extremely high
DA, Dataset-A (retaining zeros), DM, Dataset-M (zero-values imputed with means); |*r*| > 0.9 and *p* < 0.001, Almost identical; |*r*| > 0.7 and *p* < 0.001, Extremely high.			

(B) Data calibration			
Calibration anchors	Completely independent affiliation degree 0.05	Intersection point affiliation degree 0.50	Completely dependent affiliation degree 0.95
Result variables			
D6	0.200	0.800	2.670
Condition variables			
D1	0.085	0.200	0.750
D2	0.066	0.200	1.000
D3	0.066	0.249	1.088
D4	0.100	0.200	0.809
D5	0.066	0.250	1.000
D6, Social Identity; D1, Dual-dimensional perceived physical literacy kernel; D2, Dual-dimensional sense of belonging construction logic; D3, Single-dimensional inclusive leadership openness attribute; D4, Dual identity categorization conduction path; D5, Inter-group comparison output path.			

(C) Single necessary condition analysis				
Condition	Outcome: D6 (presence)		Outcome: ~D6 (absence)	
	Consistency	Coverage	Consistency	Coverage
D1	0.74	0.92	0.38	0.46
~D1	0.56	0.48	0.93*	0.78
D2	0.75	0.81	0.51	0.54
~D2	0.57	0.55	0.82	0.77
D3	0.7	0.85	0.44	0.52
~D3	0.61	0.53	0.87	0.74
D4	0.7	0.81	0.45	0.51
~D4	0.57	0.52	0.83	0.73
D5	0.76	0.69	0.66	0.58
~D5	0.53	0.62	0.65	0.73
~, absence of the condition. *, A condition is considered necessary when consistency ≥ 0.90; D6, State of group-based social identity under dual-identity context; D1, Dual-dimensional perceived physical literacy kernel; D2, Dual-dimensional sense of belonging construction logic; D3, Single-dimensional inclusive leadership openness attribute; D4, Dual identity categorization conduction path; D5, Inter-group comparison output path.				

(D) Non-necessary condition testing							
Conditions	Method	c-accuracy	Ceiling zone	Scope	Effect size(d)	*p*-value(P)	Verification
D1	CR-FDH	90.60%	0.17	0.91	0.19	0.01	No
	CE-FDH	100.00%	0.20	0.91	0.21	0.00	
D2	CR-FDH	65.60%	0.27	0.90	0.29	0.00	Yes
	CE-FDH	100.00%	0.24	0.90	0.27	0.00	
D3	CR-FDH	96.90%	0.18	0.90	0.20	0.02	No
	CE-FDH	100.00%	0.19	0.90	0.21	0.00	
D4	CR-FDH	90.60%	0.24	0.92	0.25	0.00	Yes
	CE-FDH	100.00%	0.27	0.92	0.30	0.00	
D5	CR-FDH	68.80%	0.33	0.89	0.37	0.00	Yes
	CE-FDH	100.00%	0.30	0.89	0.33	0.00	
D1, Dual-dimensional perceived physical literacy kernel; D2, Dual-dimensional sense of belonging construction logic; D3, Single-dimensional inclusive leadership openness attribute; D4, Dual identity categorization conduction path; D5, Inter-group comparison output path; Yes, *d* > 0.1 and *p* < 0.01; No, *d* < 0.1 or *p* > 0.01.							

(E) Bottleneck level testing					
D6	D1	D2	D3	D4	D5
0	NN	NN	NN	NN	NN
10	NN	NN	0.9	NN	NN
20	NN	NN	5.7	NN	3.4
30	3.8	NN	10.4	6.8	14
40	10.4	8.7	15.2	15.1	24.7
50	17	22	20	23.5	35.3
60	23.6	35.2	24.8	31.8	45.9
70	30.2	48.5	29.5	40.1	56.5
80	36.8	61.7	34.3	48.5	67.2
90	43.4	75	39.1	56.8	77.8
100	50.1	88.2	43.8	65.1	88.4
D6, State of group-based social identity under dual-identity context, Outcome Level; D1, Dual-dimensional perceived physical literacy kernel; D2, Dual-dimensional sense of belonging construction logic; D3, Single-dimensional inclusive leadership openness attribute; D4, Dual identity categorization conduction path; D5, Inter-group comparison output path.					

(F) Configuration path analysis								
Conditions	Outcome: D6 (presence)				Outcome: ~D6 (absence)			
	DMP.1	DMP.2	DAP.1	DAP.2	DMP.1	DMP.2	DAP.1	DAP.2
D1		○	●	●			⊕	⊕
D2	⊕	○		⊕		⊕	⊗	⊗
D3	●	●	⊕	●	⊕	○	○	⊕
D4	⊕	○	⊕		⊗	⊗	⊗	⊗
D5	⊕	○	⊕	⊕	○	⊕	⊕	○
Raw coverage	0.37	0.52	0.57	0.61	0.58	0.34	0.70	0.75
Unique coverage	0.10	0.26	0.12	0.16	0.35	0.11	0.09	0.14
Consistency	0.85	0.97	0.97	0.97	0.79	0.76	0.78	0.78
Solution coverage	0.63		0.73		0.69		0.85	
Solution consistency	0.89		0.84		0.77		0.76	
DMP, Dataset-M Path, Dataset-M replaced all zero-coded values with the variable-specific sample mean computed across valid responses, thereby eliminating potential systematic bias introduced by missing-value markers in set-membership calibration; DAP, Dataset-A Path, Dataset-A retained the original questionnaire coding, treating zero-coded entries (unanswered or non-applicable items) as genuine observations; ●, Core condition present; ⊗, Core condition absent; ○, Peripheral condition present; ⊕, Peripheral condition absent; blank, condition is irrelevant. Intermediate solutions are reported.; D1, Dual-dimensional perceived physical literacy kernel; D2, Dual-dimensional sense of belonging construction logic; D3, Single-dimensional inclusive leadership openness attribute; D4, Dual identity categorization conduction path; D5, Inter-group comparison output path; D6, State of group-based social identity under dual-identity context. Assumption for intermediate solution: D1 is present. Frequency cutoff 2. Core conditions are derived from parsimonious solutions; peripheral conditions derive from intermediate but not parsimonious solutions.								

#### 4.2.3 Data calibration

This study strictly followed fsQCA data calibration standards to complete standardization. The completely dependent threshold (0.95) ranged from 0.75 to2.67, the intersection point threshold (0.50) ranged from 0.2 to 0.8, and the completely dependent threshold (0.05) ranged from 0.066 to 0.2. A standardized dataset suitable for configurational analysis was ultimately obtained, effectively supporting the path and configurational testing of the social identity coupling model for university students from high-level sports teams in China (Pappas and Woodside, 2021) (see Table 3B).

#### 4.2.4 Necessary condition testing

Single necessary condition testing: This study conducted single necessary condition testing on the outcome variable D6 (state of group-based social identity under dual-identity context) and the non-outcome variable ~D6 (state of group-based social identity under dual-identity context). For the positive and negated conditions of D1 (dual-dimensional perceived physical literacy kernel), D2 (dual-dimensional sense of belonging construction logic), D3 (single-dimensional inclusive leadership openness attribute), D4 (dual identity categorization conduction path), and D5 (inter-group comparison output path), only D1 (dual-dimensional perceived physical literacy kernel) with ~D6 reached the necessary condition standard (consistency coefficient = 0.93, > 0.9), while all other conditional variables had consistency coefficients between 0.38 and 0.87, failing to meet the necessary condition standard (Pappas and Woodside, 2021). It is worth further clarifying that no abnormal (nan) phenomenon appeared in the fsQCA calculation during the single necessary condition test. This appears to directly confirm the validity and rationality of the variable assignment method in this study. This also appears to preliminarily and indirectly confirm the equivalence between the selective coding results (C1–C5) and the independent variables of the structural model [D1 (dual-dimensional perceived physical literacy kernel) to D5 (inter-group comparison output path)] (see Table 3C). The testing results indicate that none of the antecedent conditions (D1-D5) in this study are necessary conditions for the outcome variable (D6). Social identity among university students from high-level sports teams in China is not determined by a single condition, but rather driven by the synergistic configuration of multiple conditions, satisfying the prerequisites for fsQCA configurational analysis.

Non-necessary condition verification: This study validated the model’s configuration path by testing for non-necessary conditions. CR and CE dual verification were applied to the five antecedent conditions D1–D5 (Tajfel and Turner, 1979). Test results showed that D1 (dual-dimensional perceived physical literacy kernel; CR *p* = 0.01, *d* > 0.1) and D3 (Single-dimensional inclusive leadership openness attribute; CR: *p* = 0.02, *d* = 0.2) did not meet non-necessary condition standards and exhibited necessary attributes; D2 (dual-dimensional sense of belonging construction logic; *p* < 0.01, *d* > 0.27), D4 (dual identity categorization conduction path, *p* = 0, *d* > 0.25), and D5 (inter-group comparison output path; *p* = 0, *d* > 0.33) all met the *d* > 0.1, *p* < 0.01 criteria as non-necessary conditions (Tajfel and Turner, 1979) (see Table 3D).

Differentiated interpretation of fsQCA and non-necessary condition verification results: There is no logical conflict between the fsQCA and non-necessary-condition verification results. fsQCA evaluates set-level necessary dependence, indicating that group-based social identity can be formed through multiple-condition combinations without mandatory reliance on any single condition. In contrast, non-necessary-condition verification captures threshold characteristics for high-level outcome values. It shows that although D1 and D3 fail this non-necessary-condition verification, they constitute essential threshold guarantees for achieving high-level group-based social identity. The two approaches complement each other and jointly verify the complex multi-factor driving mechanism of the outcome among university students from high-level sports teams in China.

Bottleneck level test results: This study conducted a five-level hierarchical condition configurational evaluation for bottleneck level testing, mapping the D6 (state of group-based social identity under dual-identity context) hierarchical gradient (0–100%) to the hierarchical performance of five antecedent conditions: D1 (dual-dimensional perceived physical literacy kernel), D2 (dual-dimensional sense of belonging construction logic), D3 (single-dimensional inclusive leadership openness attribute), D4 (dual identity categorization conduction path), and D5 (inter-group comparison output path), to identify the minimum necessary constraints of each antecedent condition when achieving different outcome levels.

NN indicates no necessary constraint for the corresponding antecedent at that outcome level. At low outcome levels (0–40%), NN appeared frequently, indicating that antecedent conditions were not mandatory and deficiencies could be compensated via alternative paths.

When the outcome D6 (state of group-based social identity under dual-identity context) reached a relatively high level of 50% or above, NN no longer appeared for any antecedent condition, and all variables exhibited clear necessary constraints: to achieve a 50% outcome level, D1 (dual-dimensional perceived physical literacy kernel), D2 (dual-dimensional sense of belonging construction logic), D3 (single-dimensional inclusive leadership openness attribute), D4 (dual identity categorization conduction path), and D5 (inter-group comparison output path) required minimum thresholds of 17.0, 22.0, 20.0, 23.5, and 35.3%, respectively (see Table 3E).

As the target outcome level continued to rise to 100%, the minimum necessary thresholds of all antecedents increased correspondingly. When achieving a 100% outcome level, D1 (dual-dimensional perceived physical literacy kernel), D2 (dual-dimensional sense of belonging construction logic), D3 (single-dimensional inclusive leadership openness attribute), D4 (dual identity categorization conduction path), and D5 (inter-group comparison output path) required minimum thresholds of 50.1, 88.2, 43.8, 65.1, and 88.4%, respectively, (Tajfel and Turner, 1979) (see Table 3E).

The above results indicate that low-level outcomes impose no rigid mandatory requirements on antecedent conditions, but to achieve high-level outcomes above the fifth tier, all antecedent conditions have irreplaceable minimum necessary thresholds, and a single condition shortfall will directly restrict the achievement of high-level outcomes.

#### 4.2.5 Configurational path results

Truth table analysis: With a frequency threshold set at 2 and a consistency threshold set at 0.8, fsQCA intermediate solution configurational analysis was conducted for both the occurrence and non-occurrence of the outcome D6 (state of group-based social identity under dual-identity context) (Pappas and Woodside, 2021).

For the occurrence of D6 (state of group-based social identity under dual-identity context), two sufficient configurations were obtained: the overall model configurational coverage was 0.63 (> 0.5), explaining nearly 63% of high social identity cases, and the overall model consistency was 0.89, reaching the ideal discrimination standard (> 0.75). Configuration 1 (~D2 × D3 × ~D4 × ~D5): core condition D3 (single-dimensional inclusive leadership openness attribute) present; peripheral conditions D2 (dual-dimensional sense of belonging construction logic), D4 (dual identity categorization conduction path), and D5 (inter-group comparison output path) absent; D1 (dual-dimensional perceived physical literacy kernel) irrelevant; consistency 0.85 (> 0.75), raw coverage 0.37. Configuration 2 (D1 × D2 × D3 × D4 × D5): core condition D3 (single-dimensional inclusive leadership openness attribute) present; all other peripheral conditions D1 (dual-dimensional perceived physical literacy kernel), D2 (dual-dimensional belongingness construction logic), D4 (dual identity categorization conduction path), and D5 (inter-group comparison output path) simultaneously present; consistency 0.97 (> 0.75), raw coverage 0.52. Common Core Condition: The presence of D3 (single-dimensional inclusive leadership openness attribute) is the cross-configurational core condition contributing to the occurrence of D6 (state of group-based social identity under dual-identity context) (see Table 3F).

For the non-occurrence of D6 (~D6), two configurations were obtained: the overall model configurational coverage was 0.69 (> 0.5), explaining nearly 69% of non-high social identity cases, and the overall model consistency was 0.77, basically reaching the ideal discrimination standard (>0.75, Pappas and Woodside, 2021). Configuration 1 (~D3 × ~D4 × D5): core condition D4 (dual identity categorization conduction path) absent; peripheral conditions D3 (single-dimensional inclusive leadership openness attribute) absent and D5 (inter-group comparison output path) present; D1 (dual-dimensional perceived physical literacy kernel) and D2 (dual-dimensional sense of belonging construction logic) irrelevant; consistency 0.79 (> 0.75), raw coverage 0.58. Configuration 2 (~D2 × D3 × ~D4 × ~D5): core condition D4 (dual identity categorization conduction path) absent; peripheral conditions D2 (dual-dimensional belongingness construction logic) and D5 (inter-group comparison output path) absent, D3 (single-dimensional inclusive leadership openness attribute) present; D1 (dual-dimensional perceived physical literacy kernel) irrelevant; consistency 0.76 (>0.75), raw coverage = 0.34. Common Core Condition: The absence of D4 (dual identity categorization conduction path) is the cross-configurational core condition causing the non-occurrence of D6 (state of group-based social identity under dual-identity context) (see Table 3F).

#### 4.2.6 Robustness test results

##### 4.2.6.1 Primary analysis dataset (Dataset-M) robustness test

This study conducted fsQCA robustness testing by raising the consistency threshold and adjusting the frequency threshold: the consistency threshold for the occurrence of the outcome variable D6 (state of group-based social identity under dual-identity context) and non-occurrence were set at 0.85, and the frequency threshold was adjusted to 3, in order to verify the stability and reliability of configurational results.

The testing results under strict thresholds showed that only the configuration with all five antecedents D1 (dual-dimensional perceived physical literacy kernel), D2 (dual-dimensional sense of belonging construction logic), D3 (single-dimensional inclusive leadership openness attribute), D4 (dual identity categorization conduction path), and D5 (inter-group comparison output path) simultaneously present could effectively explain the occurrence of D6 (state of group-based social identity under dual-identity context), with an overall solution consistency as high as 0.97 (> 0.75), indicating that multi-condition synergistic matching is a stable sufficient path driving the high-level development of D6 (state of group-based social identity under dual-identity context).

Configurational testing for the non-occurrence of D6 (state of group-based social identity under dual-identity context) still identified a stable and valid configuration, namely the combinational path of D2 (dual-dimensional sense of belonging construction logic), D3 (single-dimensional inclusive leadership openness attribute), and D4 (dual identity categorization conduction path) absent with D5 (inter-group comparison output path) present. This configuration had an overall solution consistency of 0.84 (>0.75), and this model path also demonstrated good reliability (Pappas and Woodside, 2021).

##### 4.2.6.2 Cross-dataset (Dataset-A) robustness test

In the configurational analysis for the presence of D6 (state of group-based social identity under dual-identity context), the primary dataset Dataset-M identified two configurational paths leading to high-level social identity, both satisfying the predefined criteria. Dataset-M path 1 was the inclusive leadership single-driven type, and Dataset-M path 2 was the physical literacy- inclusive leadership comprehensive empowerment type. The alternative measurement Dataset-A similarly identified two paths, both with a consistency of 0.97 (>0.75), a solution consistency of 0.84 (>0.75), and a solution coverage of 0.73 (>0.5). Dataset-A path1, labeled the physical literacy-driven–inclusive leadership-blocked type, had D1 (dual-dimensional perceived physical literacy kernel) present and D3 (single-dimensional inclusive leadership openness attribute) absent as core conditions, with D4 (dual identity categorization conduction path) and D5 (inter-group comparison output path) peripherally absent and D2 (dual-dimensional sense of belonging construction logic) irrelevant; it achieved a raw coverage of 0.57, unique coverage of 0.12, and consistency of 0.97. Dataset-A path 2, labeled the physical literacy–inclusive leadership dual-driven type, had D1 (dual-dimensional perceived physical literacy kernel) present and D3 (single-dimensional inclusive leadership openness attribute) present as core conditions, with D2 (dual-dimensional sense of belonging construction logic) and D5 (inter-group comparison output path) peripherally absent and D4 (dual identity categorization conduction path) irrelevant; it achieved a raw coverage of 0.61, unique coverage of 0.16, and consistency of 0.97. The cross-dataset common core condition was D3 (single-dimensional inclusive leadership openness attribute) presence.

In the configurational analysis for ~D6 (failure to form the state of group-based social identity under dual-identity context), the primary dataset Dataset-M identified two configurational paths leading to social identity absence, both satisfying the prespecified criteria but approaching the threshold. Dataset-M path 1 was the identity categorization blocking type I, and Dataset-M path 2 was the identity categorization blocking type II. The two paths shared a single D4 (dual identity categorization conduction path) -absent core, with the peripheral states of D3 (single-dimensional inclusive leadership openness attribute) and D5 (inter-group comparison output path) exhibiting complementary reversal, constituting two peripheral variants of the same core structure. The alternative measurement Dataset-A identified two paths, both with a consistency of 0.78 (>0.75), a solution consistency of 0.76 (>0.75), and a solution coverage of 0.85 (>0.5, the highest across all datasets). Both Dataset-A path 1 (sense of belonging – identity categorization dual blocking type I) and Dataset-A path 2 (sense of belonging – identity categorization dual blocking type II) had D2 (dual-dimensional sense of belonging construction logic) absent and D4 absent as core conditions; in Dataset-A path 1, D3 (single-dimensional inclusive leadership openness attribute) was peripherally present and D5 (inter-group comparison output path) peripherally absent, whereas in Dataset-A path 2, D3 (single-dimensional inclusive leadership openness attribute) was peripherally absent and D5 (inter-group comparison output path) peripherally present, similarly constituting two peripheral variants of the D2 (dual-dimensional sense of belonging construction logic) and D4 (dual identity categorization conduction path) dual-absent core. They achieved raw coverages of 0.70 and 0.75, unique coverages of 0.09 and 0.14, and consistencies of 0.78 for both. The cross-dataset common core condition was D4 (dual identity categorization conduction path) absence.

Cross-dataset robustness checks were conducted from two dimensions: core condition retention and configurational structure correspondence. Regarding core conditions, the cross-dataset stable core condition for D6 (state of group-based social identity under dual-identity context) presence was D3 (single-dimensional inclusive leadership openness attribute) presence, and for ~D6 (state of group-based social identity under dual-identity context) absence was D4 (dual identity categorization conduction path) absence; both appeared as core conditions across the two datasets, indicating that the core findings are robust across measurement specifications. D1 (dual-dimensional perceived physical literacy kernel) was upgraded to a core condition for D6 (state of group-based social identity under dual-identity context) presence in Dataset-A but was only peripherally present or irrelevant in Dataset-M; D2 (dual-dimensional sense of belonging construction logic) was upgraded to a core absent condition for ~D6 (state of group-based social identity under dual-identity context) absence in Dataset-A but was only peripherally absent or irrelevant in Dataset-M. This finding is consistent with the D2 (dual-dimensional sense of belonging construction logic)–D6 (state of group-based social identity under dual-identity context) correlation test results: in Dataset-A the two showed a significant moderate positive correlation (*r* = 0.484, *p* = 0.005), whereas in Dataset-M the correlation decreased and became non-significant (*r* = 0.292, *p* = 0.105), indicating that mean-replacement treatment of zero-coded values stripped D2 (dual-dimensional sense of belonging construction logic) from a core condition to a peripheral condition, thereby more precisely distinguishing the true association between D2 (dual-dimensional sense of belonging construction logic) and D6 (state of group-based social identity under dual-identity context). Regarding configurational structure, for D6 (state of group-based social identity under dual-identity context) presence, Dataset-M path 1 and Dataset-A path 2 exhibited a superset correspondence [core D3 presence retained, with Dataset-A path 2 adding D1 (dual-dimensional perceived physical literacy kernel) core presence], indicating high robustness; Dataset-M path 2 and Dataset-A path 1 had no direct correspondence [D3 (single-dimensional inclusive leadership openness attribute) reversed from core present to core absent], indicating that this path was not robust across datasets. For ~D6 (state of group-based social identity under dual-identity context) absence, both Dataset-M path 1–Dataset-A path 1 and Dataset-M path 2–Dataset-A path 2 exhibited correspondences with core D4 (dual identity categorization conduction path) absence retained and D2 (dual-dimensional sense of belonging construction logic) role enhanced, indicating basic robustness. Moreover, the two paths within Dataset-M shared a single D4 (dual identity categorization conduction path)-absent core, and the two paths within Dataset-A shared a D2 (dual-dimensional sense of belonging construction logic) and D4 (dual identity categorization conduction path) dual-absent core, with D3 (single-dimensional inclusive leadership openness attribute) and D5 (inter-group comparison output path) exhibiting complementary reversal patterns within each dataset, indicating that zero-coding treatment did not alter the fundamental structural logic of the configurations. Regarding overall solution metrics, all eight individual configuration consistencies and four solution-level consistencies exceeded 0.75, satisfying the criterion; the ~D6 (state of group-based social identity under dual-identity context) solution consistencies (0.76–0.77) approached the threshold and should be noted in the discussion. Dataset-A achieved higher coverage [D6 (state of group-based social identity under dual-identity context): 0.73 vs. 0.63; ~D6 (state of group-based social identity under dual-identity context): 0.85 vs. 0.69] but identified more core conditions; Dataset-M had more parsimonious core conditions and more conservative, reliable results.

## 5 Discussion

### 5.1 Theoretical analysis of the social identity coupling model

This study is grounded in the dual-career preparation roles of university students from high-level sports teams in China, takes perceived physical literacy as the perceptual-behavioral logical origin, and relies on grounded theory and fsQCA configurational analysis to construct a “categorization-identification-comparison” social identity coupling model. Based on qualitative coding results (172 initial concepts, 36 axial categories, 5 core categories) and empirical outputs (necessary condition testing, configurational paths, reliability and bottleneck level testing), this section analyzes the model’s internal structure, variable mechanisms, and theoretical assumptions.

The social identity coupling model theoretically extends the classic logic of social identity theory while embedding sports-specific physical perception variables: inclusive leadership is set as the organizational environmental antecedent, the dual-dimensional perceived physical literacy kernel serves as the individual perceptual origin, belongingness undertakes the emotional mediating function, and dual identity categorization and inter-group comparison jointly constitute the social identity core. Grounded theory saturation testing confirmed that coding categories can majority cover real psychological phenomena of university students from high-level sports teams in China, including identity cognition, study-training conflict, intra-team interpersonal relations, campus cross-group socialization, physical experience, and institutional perception, providing qualitative evidence for the model. However, fsQCA results indicated that the variables in the presupposed structural model may not follow stable linear progressive relationships in real contexts.

Regarding the differentiated functions of variables, perceived physical literacy exhibits a typical “risk-prevention rather than promotion” effect. Existing studies predominantly focus on the positive promotional functions of physical literacy on physical activity and mental health among ordinary youth (Fu et al., 2025a). This is similar to the finding in this study that the absence of physical literacy [D1 (Dual-dimensional perceived physical literacy kernel)] is a necessary condition for the absence of D6 (State of group-based social identity under dual-identity context). Empirical research (Fu et al., 2025b) and this study both indicate that physical literacy is a multi-dimensional subjective perceptual construct encompassing not only physical fitness and athletic competence but also motivation, physical self-perception, and movement knowledge understanding, and individual physical perception further shapes social psychological processing in group contexts. Studies on competitive sports participants further suggest that athletes’ physical literacy perception differs from the general population (Fu et al., 2026). The results of this study’s five-level bottleneck analysis indicate that training stress and experiences with sports injuries significantly alter individuals’ subjective evaluations of their bodies, and a decline in perceived physical fitness can trigger negative emotions and reduce willingness to participate in group activities. Large-scale epidemiological studies have confirmed significant correlations between physical literacy perception and individual belongingness/group attachment, consistent with the findings of this study; but only verified linear correlations without revealing the asymmetric causal characteristics of physical literacy in group identity formation (Diao et al., 2024). Public health research emphasizes the intervention value of physical literacy, regarding its enhancement as an entry point for improving individual mental health (Yan et al., 2022); however, like this study, that study also fails to distinguish between the two distinct mechanisms of action: “risk avoidance” and “positive development.”

Sense of belonging may act as a pivotal intermediary hub in the coupling model. Robust configurational results indicate that the presence of D3 (single-dimensional inclusive leadership openness attribute) is the cross-configurational core condition contributing to the occurrence of D6 (state of group-based social identity under dual-identity context). Existing research on a sense of belonging, as well as this study, generally confirms that a sense of belonging is the core emotional carrier of individual-to-group psychological transition (Sotardi and Rudolf, 2026). Campus group context research (Sotardi and Rudolf, 2026) and the D2 (Dual-dimensional sense of belonging construction logic) analysis in this study both indicate that belongingness is the subjective experience of feeling accepted by the group and obtaining emotional bonds, serving as a key mediating variable connecting individual cognition and group identity. Research on adolescent athletes further proposes that belongingness within sports teams derives not solely from training performance but also from role positioning, peer support, and identity integration, which highly aligns with the dual-role reality of university students from high-level sports teams in China in this study (Allen et al., 2021). Belongingness theoretical reviews emphasize its “bidirectional transmission” characteristic; however, these reviews are largely based on traditional linear assumptions and may not examine configurational effects under multi-condition concurrency (Allen et al., 2021). Both the external environment and individual cognition can influence a sense of belonging, which in turn shapes group attitudes and behavioral outcomes, as illustrated by the chain structure in this study: A148 (willingness to voluntarily sign up for a fun sports meet organized by a general student organization) → B7 (Dual identity conflict experience) → C/D2 (Dual-dimensional sense of belonging construction logic). Large-scale empirical studies verify the positive predictive effect of belongingness on group identity, but treat belongingness as a continuous gain variable, assuming higher belongingness necessarily leads to higher social identity, potentially lacking discussion of the negative configurational mechanism of belongingness deficiency (Ge et al., 2026). The two configurations examined in this study (~D2 × D3 × ~D4 × ~D5, ~D2 × D3 × ~D4 × ~D5) may serve as a useful complement to the negative configuration mechanism underlying a lack of sense of belonging. Belongingness research from an identity conflict perspective suggests that when individuals face multiple role expectations simultaneously, the mediating effect of belongingness is moderated by identity tension, and multiple identity conflicts weaken the transformation efficiency of belongingness, providing theoretical reference for explaining the uniqueness of this study’s sample (Slaten et al., 2016).

Inclusive leadership functions as a critical external organizational antecedent with core configurational contribution in high social identity paths but have no single independent necessary condition. Classic research indicates that the core of inclusive leadership lies in leaders’ open listening, acceptance of subordinates’ viewpoints, creation of a safe interactive atmosphere, and promotion of members’ organizational involvement (Al-Atwi and Al-Hassani, 2021). Four sub-dimensions of D3 (Single-dimensional inclusive leadership openness attribute) in this study—including B3 (Perception of organizational inclusive behavior), B15 (Perception of differentiated support), B24 (Perception of evaluation fairness), and B31 (Positive perception of inclusive climate)—appear to confirm the core attributes of classical inclusive leadership. Empirical research in sports team contexts demonstrates that coaches’ inclusive behaviors positively predict athletes’ psychological safety and group attachment. These studies mostly migrate enterprise organizational frameworks to sports contexts, verifying positive aftereffects of inclusive leadership but primarily using linear regression to test net effects (King et al., 2024). The chain structure in this study—A31 (Positive perception of an inclusive atmosphere from coaches) to B31 (Positive perception of inclusive climate)—also appears to confirm the positive spillover phenomenon of inclusive leadership. Recent inclusive leadership research further distinguishes its dual effects: inclusive leadership can produce promotion effects but also has boundary conditions—when individual internal psychological resources are insufficient, organizational-level inclusiveness is difficult to transform into positive individual psychological outcomes. However, this research may not have revealed multi-condition concurrent mechanisms from a configurational perspective (Yoon and Hong, 2026). The four configuration paths in this study that include inclusive leadership [D3 (Single-dimensional inclusive leadership openness attribute)] appear to be a useful addition for further distinguishing the dual effects of inclusive leadership. Research on dual identity conflict groups suggests that the effect of inclusive leadership on group identity is constrained by individual identity processing, and organizational inclusiveness cannot directly resolve internal identity contradictions (Nguyen et al., 2024). Existing literature universally assumes that higher levels of inclusive leadership will continuously yield better group psychological outcomes (Al-Atwi and Al-Hassani, 2021; King et al., 2024; Yoon and Hong, 2026; Nguyen et al., 2024), implying a promotion assumption consistent with this study’s findings.

This study’s fsQCA results potentially supplement existing literature: the coexistence of high inclusive leadership and non-high social identity in some samples indicates that organizational inclusiveness cannot fully offset the negative effects of individual perceptual defects and identity conflict, revealing possible fractures in the chain transmission path.

### 5.2 Sensitivity characteristics of the social identity structure model

By constructing two parallel datasets (Dataset-A and Dataset-M) and conducting a multi-evidence sensitivity analysis, this study systematically evaluated the potential impact of zero-coding treatment on fsQCA configurational outcomes. We found that although replacing zero-coded values with sample means produced limited perturbation at the case level (Pearson r = 0.944–0.993), it did not alter the relative ranking of cases along each antecedent condition dimension. At the configurational level, core causal pathways and core conditions remained stable across the two datasets, indicating that the dual-identity social identity coupling configurations identified in this study exhibit robustness across alternative measurement specifications. This finding carries methodological implications for fsQCA applications in the social identity domain: given the prevalence of zero-coded entries in questionnaire data (e.g., unanswered items, non-applicable responses, initial values of reverse-coded items), researchers should carefully assess the impact of missing markers on set-membership calibration and verify the stability of configurational conclusions through multi-dataset comparisons rather than relying on a single coding scheme.

The change in the correlation between belongingness (D2) and social identity (D6) observed at the construct level warrants in-depth discussion. In Dataset-A, the two constructs showed a significant moderate positive correlation (*r* = 0.484), whereas in Dataset-M, where zero-coded values were replaced by means, the correlation decreased to 0.292 and was no longer significant. This result carries at least two theoretical implications. First, belongingness and social identity do exhibit conceptual overlap—belongingness emphasizes emotional attachment and perceived acceptance by the group, while social identity emphasizes cognitive awareness and value commitment to group membership, and the two inevitably share semantic space in measurement items. When shared zero-coding patterns exist in the data, this conceptual overlap is artificially amplified, manifesting as inflated correlation coefficients. Second, the decrease in correlation after mean replacement does not imply that the two constructs are unrelated; rather, it suggests that the true magnitude of their association may be lower than that indicated by the original coded data. Within the fsQCA framework, this finding is particularly meaningful: fsQCA focuses on configurational effects among conditions rather than bivariate net correlations, so even if the bivariate correlation between D2 and D6 fluctuates across coding schemes, their complementary or substitutive roles within configurations may remain stable—a proposition confirmed by our configurational-level results.

From a practical perspective, the sensitivity analysis results of this study provide a more reliable evidence base for intervention practices targeting the dual-identity social identity coupling model among Chinese high-level athletic team university students. Because the core configurations remained stable across the two datasets, the condition combinations identified in this study that lead to high-level social identity coupling (e.g., the “inclusive leadership single-driven type” configuration, i.e., D3 single-dimensional inclusive leadership openness attribute as the core driver) exhibit cross-measurement consistency, meaning that intervention strategies designed based on these configurations (e.g., targeted interventions focusing on the D3 single-dimensional inclusive leadership openness attribute) are unlikely to fail due to differences in data coding methods. Furthermore, the changes in the correlation coefficient between D2 and D6 across coding schemes suggest that in intervention practices targeting belongingness and social identity, researchers should distinguish between their independent and joint effects, avoiding misinterpreting spurious covariation caused by conceptual overlap as causal associations. Future intervention studies may adopt longitudinal designs to separately track the independent change trajectories of belongingness and social identity, thereby more precisely revealing their mechanisms of action in the dual-identity coupling process.

### 5.3 Practical intervention strategies

Based on fsQCA configurational paths, bottleneck level hierarchical testing, and grounded theory coding results, combined with frontier social identity theory research (Zhang and Li, 2022; Williams, 2023; Koo et al., 2025), this study hierarchically constructs organizational psychological cultivation practical schemes adapted to university students from high-level sports teams in China, and proposes three hierarchical intervention strategies for social identity enhancement.

#### 5.3.1 Bottom-line prevention intervention for perceived physical literacy

Targeted hierarchical physical intervention schemes were designed for the dual scenarios of athletic training and academic pressure, focusing on eliminating the risk of non-high social identity induced by insufficient physical literacy perception and potentially filling the intervention gap for dual-role student-athletes.

#### 5.3.2 Hub mediation intervention for group belongingness

Compared with pure online community belongingness construction (Koo et al., 2025) and activity programs targeting single social movement groups (Ai and Tanimoto, 2026), this strategy may adapt to the dual “athletic + campus” fields of university sports teams, takes belongingness as the core to resolve dual identity categorization conflicts, and alleviates the belonging fragmentation caused by dual identity.

#### 5.3.3 Environmental empowerment intervention of inclusive leadership

Breaking the limitations of single linear organizational intervention, this study may construct compound inclusive leadership management strategies to build a long-term positive social identity organizational environment and realize sustained psychological empowerment of student-athletes (Koo et al., 2025; Ai and Tanimoto, 2026).

### 5.4 Strengths and limitations

#### 5.4.1 Strengths

Theoretically, this study innovatively incorporates perceived physical literacy into the social identity antecedent system, is grounded in the dual-role attributes of university students from high-level sports teams in China, potentially constructs a localized social identity structural model, compensates for the insufficiency of traditional social identity research in paying attention to physical perception of sports groups, and expands the explanatory boundary of social identity theory in university competitive sports contexts.

Methodologically, the mixed-method design combining grounded theory and fsQCA effectively integrates qualitative theoretical construction and quantitative configurational verification, perhaps breaking the net effect limitation of traditional linear regression and fitting the complex nonlinear psychological mechanism of the research population.

Practically, this study perhaps distinguished the risk-prevention and promotion functions of core variables through bottleneck hierarchical analysis, providing refined, operable hierarchical intervention logic for university sports team psychological management and talent cultivation.

#### 5.4.2 Limitations

First, regarding the sample, this study included 32 main samples for grounded theory and 4 saturation test samples, satisfying the grounded theory saturation standard. However, the sample distribution has limitations in terms of region, sport event, and athletic level. Future research can expand the sample scope to further verify the external validity of the model.

Second, there is a simplification phenomenon in variable processing. This study treated inclusive leadership as a unidimensional condition for configurational calculation, but qualitative coding revealed that coach’s inclusive behaviors encompass multiple connotations including training support, academic assistance, and psychological care, and the unidimensional setting masks the differentiated configurational effects of different inclusive behaviors.

Additionally, structural factors such as the study-training system, training arrangements, and academic assessment frequently appeared in open coding, but were not included as explicit variables in fsQCA analysis and served only as background factors. Consequently, the model has limited direct explanatory power for institutional structural constraints.

Admittedly, despite the limited overall perturbation, the altered association between D2 and D6 indicates that the re-assignment procedure is not entirely unbiased. Findings regarding dual-identity configurations should be interpreted with caution concerning the influence of zero-value treatment.

Regarding missing value (zero-value) handling, although this study constructed two parallel datasets (Dataset-A and Dataset-M), conducted multi-evidence sensitivity analysis, and combined qualitative coding evidence for triangulated interpretation, thereby verifying the robustness of core configurational conclusions to zero-coding treatment methods, mean replacement (Dataset-M) itself, as a missing value imputation method, still has inherent limitations: mean replacement compresses the variance of variables, potentially underestimating the true degree of variation among conditions, thereby exerting a slight impact on the discrimination of fuzzy-set calibration. In addition, this study did not attempt other missing value handling methods (e.g., multiple imputation, maximum likelihood estimation, K-nearest neighbor imputation), and thus cannot rule out the possibility that other imputation strategies may lead to changes in configurational results. Future research may, on the basis of larger samples, systematically compare the effects of multiple missing value handling methods on fsQCA configurational results, thereby further establishing best practice norms for fsQCA research in the field of social identity.

Finally, this study is cross-sectional research that can only identify concurrent configurational relationships among conditions and cannot strictly verify dynamic temporal causality among variables. The temporal sequence assumption of the chain model still requires further verification through longitudinal tracking data.

## 6 Conclusion

### 6.1 Core conclusion

Taking the dual-career preparation attributes of university students from high-level sports teams in China as the research entry point and perceived physical literacy as the perceptual-behavioral logical origin, this study constructed and empirically verified a five-dimensional localized social identity structural model via grounded theory and fsQCA mixed methods.

First, this study extracted 172 initial concepts, 36 axial categories, and 5 core selective coding categories with verified theoretical saturation, constructing a dual-role adapted social identity coupling model that inherits classic theoretical connotations and potentially fits the localized psychological characteristics of Chinese high-level student-athletes.

Second, the social identity formation of university students from high-level sports teams in China follows multi-condition configurational driving logic without single independent necessary conditions, presenting significant asymmetric causal characteristics between high and non-high social identity paths. Perceived physical literacy predominantly undertakes risk-prevention functions, and sense of belonging may serve as the pivotal intermediary connecting individual perception and group identity.

Third, bottleneck level testing verified the transmission fracture risk of configurational paths. As a core organizational antecedent, inclusive leadership potentially provides a favorable situational foundation for high-level social identity but may not fully offset negative psychological outcomes caused by individual perceptual defects and dual identity conflict.

### 6.2 Social and theoretical value

Theoretically, this study pioneers the integration of perceived physical literacy into the social identity theoretical framework, potentially expands the application scenarios of social identity theory in university competitive sports, enriches localized empirical evidence of organizational psychology among university students from high-level sports teams in China, and provides a referable analytical framework for subsequent related research.

Practically, current Chinese university high-level sports team construction must balance athletic talent cultivation with student-athletes’ academic development, and the psychological conflict perhaps caused by dual roles is a practical issue that cannot be ignored in sports team management.

This study identified the differentiated functions of various psychological conditions and proposed three hierarchical intervention strategies for social identity enhancement, potentially providing empirical support for university sports team coaches and managers in psychological construction, organizational atmosphere optimization, and study-training identity conflict alleviation, contributing to the comprehensive development and high-quality talent training of high-level student-athletes in Chinese universities.

## Data Availability

The original contributions presented in the study are included in the article/Supplementary material, further inquiries can be directed to the corresponding author/s.
