# Supplementary material for: Social identity coupling model in university students in high-level sports teams: based on grounded theory and fsQCA
Source: Front Psychol. 2026 Oct 5;17:1971252. doi: 10.3389/fpsyg.2026.1971252 (PMC13677527; doi:10.3389/fpsyg.2026.1971252)
Supplement: Supplementary file 1 [file Table_1.DOCX]

Supplementary Material

# 1 Supplementary Table 1

**TABLE 1. Demographic Characteristics of University Students from High-Level Sports Teams in China.**

| **Item** | **Target Sample - N** | **Target Sample - %** | **Random Sample - N** | **Random Sample - %** |
| --- | --- | --- | --- | --- |
| Gender |  |  |  |  |
| Female | 35 | 46.05% | 24 | 66.67% |
| Male | 41 | 53.95% | 12 | 33.33% |
| Age |  |  |  |  |
| 18-19 years old | 32 | 42.11% | 10 | 27.78% |
| 20-21 years old | 16 | 21.05% | 12 | 33.33% |
| 22 years old and above | 28 | 36.84% | 14 | 38.89% |
| University |  |  |  |  |
| Project 985 University | 15 | 19.74% | 5 | 13.89% |
| Project 211 University | 22 | 28.95% | 11 | 30.56% |
| Double First-Class University | 26 | 34.21% | 13 | 36.11% |
| Comprehensive University | 13 | 17.11% | 7 | 19.44% |
| Grade |  |  |  |  |
| Grade 1 | 28 | 36.84% | 13 | 36.11% |
| Grade 2 | 30 | 39.47% | 15 | 41.67% |
| Grade 3 | 11 | 14.47% | 6 | 16.67% |
| Grade 4 | 7 | 9.21% | 2 | 5.56% |
| Academic Major |  |  |  |  |
| Science | 24 | 31.58% | 8 | 22.22% |
| Engineering | 21 | 27.63% | 11 | 30.56% |
| Arts and Humanities | 31 | 40.79% | 17 | 47.22% |
| Degree |  |  |  |  |
| Bachelor | 42 | 55.26% | 22 | 61.11% |
| Master | 31 | 40.79% | 12 | 33.33% |
| Doctor | 3 | 3.95% | 2 | 5.56% |
| Sport Event |  |  |  |  |
| Gymnastics | 25 | 32.89% | 6 | 16.67% |
| Ball Games | 32 | 42.11% | 16 | 44.44% |
| Athletics | 8 | 10.53% | 7 | 19.44% |
| Swimming | 6 | 7.89% | 5 | 13.89% |
| Other Sports | 5 | 6.58% | 2 | 5.56% |
| Athletic Level |  |  |  |  |
| National Level 1 Athlete | 46 | 60.53% | 21 | 58.33% |
| National Master of Sports | 28 | 36.84% | 14 | 38.89% |
| International Master of Sports | 2 | 2.63% | 1 | 2.78% |
