# Supplementary material for: Social identity coupling model in university students in high-level sports teams: based on grounded theory and fsQCA
Source: Front Psychol. 2026 Oct 5;17:1971252. doi: 10.3389/fpsyg.2026.1971252 (PMC13677527; doi:10.3389/fpsyg.2026.1971252)
Supplement: Supplementary file 2 [file Table_2.DOCX]

Supplementary Material

# 1 Supplementary Table 2

**TABLE 2. Statistical table of data based on grounded theory.**

(A) **Semi-Structured Interview for the Social Identity Model**

| **Dimensions** | **Description** |
| --- | --- |
| I. Physical Literacy (Variable 1) | |
| Dimension 1:  Motivation and Confidence | 1. When participating in collective or group-based physical activities, what is your willingness to get involved? What factors facilitate or constrain your motivation to participate? |
|  | 2. For group tasks that demand physical engagement, how confident are you about your performance? How does this confidence level influence your participation and behavior within the group? |
| Dimension 2:  Physical Competence | 1. To what extent do your basic physical fitness, motor skills and physical adaptability meet the practical requirements of routine group activities and collective tasks? Please illustrate with specific examples. |
|  | 2. How do your strengths or limitations in physical competence affect your group integration and teamwork when activities involve physical demands? |
| Dimension 3:  Knowledge and understanding | 1. To what extent do you possess knowledge about scientific exercise, physical conditioning and health protection? How does such knowledge guide your engagement in group-based physical activities? |
|  | 2. How does your awareness of your physical status, physical patterns and physical limitations shape your self-positioning, participation choices, and subjective experience of social identity within the group? Please elaborate. |
| II. Sense of Belonging (Variable 2) | |
| Dimension 4:  Social Identity | 1. To what extent do you recognize and accept your membership in this group? How does this group-member identity shape your behaviors and attitudes within the collective? |
|  | 2. Compared with external groups, do you develop a distinct sense of group identity for your in-group? What behaviors reflect this group-based social identity? |
| Dimension 5:  Belonging | 1. During daily interactions and group activities, to what extent do you feel accepted and included by the group? How does this perceived acceptance reinforce your sense of belonging? |
|  | 2. Do you feel genuinely integrated and sustain a stable sense of belonging in this group? Have you experienced marginalization or a lack of belonging? |
| Dimension 6:  Attachment | 1. Over time, have you developed emotional attachment toward the group and its members? In what situations or observable ways is this attachment manifested? |
|  | 2. If you were to leave or switch groups, would you experience feelings of reluctance? How does this group attachment influence your social identity and willingness to participate? |
| III. Inclusive Leadership (Variable 3) | |
| Dimension 7:  Openness | 1. In routine group operations and activities, to what extent do leaders maintain an open-minded stance toward diverse viewpoints, new ideas and dissenting opinions from members? Please provide concrete examples. |
|  | 2. When members propose suggestions inconsistent with existing group decisions or leaders’ preferences, how do leaders typically respond? To what extent are divergent perspectives respected and incorporated? How does such openness affect your sense of engagement? |
| Dimension 8:  Availability | 1. When you encounter difficulties or confusion in group-related affairs, are leaders able to offer timely resources, assistance and guidance? How sufficient and relevant is the support provided? |
|  | 2. To what extent do leaders’ support and available resources match members’ practical needs? How do these available supports influence your recognition and sense of belonging toward the group? |
| Dimension 9:  Accessibility | 1. How accessible and approachable do you perceive your leaders to be? Can you comfortably and efficiently raise concerns, express needs and communicate ideas with them? |
|  | 2. How does leader accessibility influence your participation motivation, emotional investment and group-based social identity? What subjective differences do you perceive between approachable versus distant leadership styles? |
| IV. Supplementary Open-ended Questions | |
|  | 1. Based on your experience, to what extent do physical literacy, sense of belonging and inclusive leadership interact with one another? Please describe these inter-relationships. |
|  | 2. Beyond the themes discussed above, what additional factors may shape your social identity within the group? |
|  | 3. What adjustments to group climate, leadership practices or individual conditions could enhance members’ social identity and sense of belonging? |

(B) **Open coding**

| **Code** | **Label** |
| --- | --- |
| A1 | Perception of Sports Team Identity Label |
| A2 | Frequency of Daily Training Participation |
| A3 | Perception of Coaches' Inclusive Behaviors |
| A4 | Difficulty in Integrating into Campus Courses |
| A5 | Frequency of Teammate Interaction |
| ------ | ------ |
| A169 | Experience of Humanistic Care from Coaches' Proactive Attention to Athletes' Daily Emotional State |
| A170 | Perception of Identity Transition Support from School-arranged Pairing Assistance During the Retirement Transition Period |
| A171 | Public Affairs Participation Behavior in Proactively Participating in the Management of Sports Team Student Associations |
| A172 | Perception of the Rules of the Dual Graduation Criteria for Training and Academics Implemented by Sports Teams |
| Examples | |
| Interview | II. Sense of Belonging (Independent Variable 2)  1. During daily interactions and group activities, to what extent do you feel accepted and included by the group? How does this perceived acceptance reinforce your sense of belonging? |
| Answer | 1. As elite student‑athletes on the university team, we are exempted from some general compulsory courses and are not required to earn full credits for these courses like ordinary students, which represents a preferential policy exclusively offered to student‑athletes by the university.  2. I can clearly perceive this special treatment, which alleviates academic pressure caused by time conflicts with training and competitions and allows us to devote more time to specialized athletic training. |
| Code | 1. Coding anchoring: The two sentences consist of three components: (1) subjects: high‑level student‑athletes; (2) objective institutional policy: exemption from selected general compulsory courses; (3) core construct: individuals’ subjective perceptions of preferential institutional arrangements.  2. Text interpretation: The first quote presents factual institutional context and contrasts credit requirements between student‑athletes and regular undergraduates. The second quote captures subjective experience, illustrating how the policy mitigates academic‑training conflicts and operationalizes the core “perception” element of this code.  3. Open‑coding node: A105 Perceptions of special treatment granted to athletes through exemptions from certain general education courses.  4. Fit justification: The two excerpts establish a logical sequence from institutional reality to subjective perception, fully capturing the meaning scope of code A105. The material is suitable as raw quotation for open coding in grounded‑theory analysis. |

(C) **Axial coding**

| **Code** | **Label** |
| --- | --- |
| B1 | Perception of In-group Identity Categorization |
| B2 | Characteristics of Training Behavior Engagement |
| B3 | Perception of Organizational Inclusive Behaviors |
| B4 | Characteristics of Campus Academic Adaptation |
| B5 | Quality of In-group Interaction |
| ------ | ------ |
| B34 | Perception of Barriers to Physical Literacy Development |
| B35 | Experience of Lack of Inclusive Climate |
| B36 | Perception of Dual-Identity Synergy |

(D) **Selective coding and model dimensions**

| **Selective Code** | **Model Dimensions** | **Label** |
| --- | --- | --- |
| C1 | D1 | Dual-dimensional perceived physical literacy kernel |
|  | B6 | Self-perception of physical literacy |
|  | B10 | Characteristics of daily exercise behavior |
|  | B18 | Self-assessment of motor skills |
|  | B30 | Autonomous exercise planning ability |
| C2 | D2 | Dual-dimensional sense of belonging construction logic |
|  | B7 | Dual identity conflict experience |
|  | B11 | Dual identity belonging experience |
|  | B13 | Perception of athletic identity |
|  | B29 | Perception of student identity |
| C3 | D3 | Single-dimensional inclusive leadership openness attribute |
|  | B3 | Perception of organizational inclusive behavior |
|  | B15 | Perception of differentiated support |
|  | B24 | Perception of evaluation fairness |
|  | B31 | Positive perception of inclusive climate |
| C4 | D4 | Dual identity categorization conduction path |
|  | B1 | Perception of in-group identity categorization |
|  | B9 | Degree of organizational rule cognition |
|  | B21 | Degree of in-group identity consensus |
| C5 | D5 | Inter-group comparison output path |
|  | B5 | Quality of in-group interaction |
|  | B16 | Willingness for cross-group interaction |
|  | B27 | Positive experience of intergroup comparison |
|  | B33 | Negative experience of intergroup comparison |
| C6 | D6 | State of group‑based social identity under dual‑identity context |
|  | B12 | Perception of group‑based academics‑training conflict |
|  | B19 | Negative experience of group‑based identity rejection |
|  | B23 | Experience of group‑based belonging‑deficit |
|  | B32 | Characteristics of group‑based campus social integration |
|  | B36 | Perception of group‑based dual‑identity synergy |
