# Supplementary material for: Social identity coupling model in university students in high-level sports teams: based on grounded theory and fsQCA
Source: Front Psychol. 2026 Oct 5;17:1971252. doi: 10.3389/fpsyg.2026.1971252 (PMC13677527; doi:10.3389/fpsyg.2026.1971252)
Supplement: Supplementary file 3 [file Table_3.DOCX]

Supplementary Material

# 1 Supplementary Table 3

**TABLE 3.** **FsQCA of the Social Recognition Model for University students from high‑level sports teams in China.**

(A) **Sensitivity analysis of Pearson correlations across Dataset‑A (retaining zeros) and Dataset‑M (zero‑values imputed with means).**

| **Cross-Validation** | **r** | | **p** | | **Interpretation** |
| --- | --- | --- | --- | --- | --- |
| DA6 ↔ DM6 | 0.944 | p<0.001 | | Extremely high | |
| DA1 ↔ DM1 | 0.973 | | p<0.001 | | Extremely high |
| DA2 ↔ DM2 | 0.993 | | p<0.001 | | Almost identical |
| DA3 ↔ DM3 | 0.973 | | p<0.001 | | Extremely high |
| DA4 ↔ DM4 | 0.977 | | p<0.001 | | Extremely high |
| DA5 ↔ DM5 | 0.984 | | p<0.001 | | Extremely high |

**Remarks:** DA, Dataset‑A (retaining zeros), DM, Dataset‑M (zero‑values imputed with means); |r| > 0.9 and p<0.001, Almost identical；|r| > 0.7 and p<0.001, Extremely high.

(B) **Data Calibration**.

| **Calibration anchors** | **Completely independent**  **Affiliation degree 0.05** | **Intersection point**  **Affiliation degree 0.50** | **Completely dependent**  **Affiliation degree 0.95** |
| --- | --- | --- | --- |
| **Result variables** | | | |
| D6 | 0.200 | 0.800 | 2.670 |
| **Condition variables** | | | |
| D1 | 0.085 | 0.200 | 0.750 |
| D2 | 0.066 | 0.200 | 1.000 |
| D3 | 0.066 | 0.249 | 1.088 |
| D4 | 0.100 | 0.200 | 0.809 |
| D5 | 0.066 | 0.250 | 1.000 |

**Remarks:** D6, State of group‑based social identity under dual‑identity context; D1, Dual-dimensional perceived physical literacy kernel; D2, Dual-dimensional sense of belonging construction logic; D3, Single-dimensional inclusive leadership openness attribute; D4, Dual identity categorization conduction path; D5, Inter-group comparison output path.

(C) **Single necessary condition analysis**.

| **Condition** | **Outcome: D6(presence)** | | **Outcome: ~D6(absence)** | |
| --- | --- | --- | --- | --- |
|  | **Consistency** | **Coverage** | **Consistency** | **Coverage** |
| D1 | 0.74 | 0.92 | 0.38 | 0.46 |
| ~D1 | 0.56 | 0.48 | 0.93* | 0.78 |
| D2 | 0.75 | 0.81 | 0.51 | 0.54 |
| ~D2 | 0.57 | 0.55 | 0.82 | 0.77 |
| D3 | 0.70 | 0.85 | 0.44 | 0.52 |
| ~D3 | 0.61 | 0.53 | 0.87 | 0.74 |
| D4 | 0.70 | 0.81 | 0.45 | 0.51 |
| ~D4 | 0.57 | 0.52 | 0.83 | 0.73 |
| D5 | 0.76 | 0.69 | 0.66 | 0.58 |
| ~D5 | 0.53 | 0.62 | 0.65 | 0.73 |

**Remarks:** ~, absence of the condition. *, A condition is considered necessary when consistency ≥ 0.90; D6, State of group‑based social identity under dual‑identity context; D1, Dual-dimensional perceived physical literacy kernel; D2, Dual-dimensional sense of belonging construction logic; D3, Single-dimensional inclusive leadership openness attribute; D4, Dual identity categorization conduction path; D5, Inter-group comparison output path.

(D) **Non-Necessary Condition Testing**

| **Conditions** | **Method** | **c‑accuracy** | **Ceiling zone** | **Scope** | **Effect size（d）** | **p‑value(P)** | **verification** |
| --- | --- | --- | --- | --- | --- | --- | --- |
| D1 | CR‑FDH | 90.60% | 0.17 | 0.91 | 0.19 | 0.01 | No |
|  | CE‑FDH | 100.00% | 0.20 | 0.91 | 0.21 | 0.00 |  |
| D2 | CR‑FDH | 65.60% | 0.27 | 0.90 | 0.29 | 0.00 | Yes |
|  | CE‑FDH | 100.00% | 0.24 | 0.90 | 0.27 | 0.00 |  |
| D3 | CR‑FDH | 96.90% | 0.18 | 0.90 | 0.20 | 0.02 | No |
|  | CE‑FDH | 100.00% | 0.19 | 0.90 | 0.21 | 0.00 |  |
| D4 | CR‑FDH | 90.60% | 0.24 | 0.92 | 0.25 | 0.00 | Yes |
|  | CE‑FDH | 100.00% | 0.27 | 0.92 | 0.30 | 0.00 |  |
| D5 | CR‑FDH | 68.80% | 0.33 | 0.89 | 0.37 | 0.00 | Yes |
|  | CE‑FDH | 100.00% | 0.30 | 0.89 | 0.33 | 0.00 |  |

**Remarks:** D1, Dual-dimensional perceived physical literacy kernel; D2, Dual-dimensional sense of belonging construction logic; D3, Single-dimensional inclusive leadership openness attribute; D4, Dual identity categorization conduction path; D5, Inter-group comparison output path; Yes, d > 0.1 and p < 0.01; No, d < 0.1 or p > 0.01.

(E) **Bottleneck level testing**

| **D6** | **D1** | **D2** | **D3** | **D4** | **D5** |
| --- | --- | --- | --- | --- | --- |
| 0 | NN | NN | NN | NN | NN |
| 10 | NN | NN | 0.9 | NN | NN |
| 20 | NN | NN | 5.7 | NN | 3.4 |
| 30 | 3.8 | NN | 10.4 | 6.8 | 14 |
| 40 | 10.4 | 8.7 | 15.2 | 15.1 | 24.7 |
| 50 | 17 | 22 | 20 | 23.5 | 35.3 |
| 60 | 23.6 | 35.2 | 24.8 | 31.8 | 45.9 |
| 70 | 30.2 | 48.5 | 29.5 | 40.1 | 56.5 |
| 80 | 36.8 | 61.7 | 34.3 | 48.5 | 67.2 |
| 90 | 43.4 | 75 | 39.1 | 56.8 | 77.8 |
| 100 | 50.1 | 88.2 | 43.8 | 65.1 | 88.4 |

**Remarks:** D6, State of group‑based social identity under dual‑identity context, Outcome Level; D1, Dual-dimensional perceived physical literacy kernel; D2, Dual-dimensional sense of belonging construction logic; D3, Single-dimensional inclusive leadership openness attribute; D4, Dual identity categorization conduction path; D5, Inter-group comparison output path.

(F) **Configuration path analysis**.

| **Conditions** | **Outcome: D6 (presence)** | | | | **Outcome: ~D6 (absence)** | | | |
| --- | --- | --- | --- | --- | --- | --- | --- | --- |
|  | **DMP.1** | **DMP.2** | **DAP.1** | **DAP.2** | **DMP.1** | **DMP.2** | **DAP.1** | **DAP.2** |
| D1 |  | ○ | ● | ● |  |  | ⊕ | ⊕ |
| D2 | ⊕ | ○ |  | ⊕ |  | ⊕ | ⊗ | ⊗ |
| D3 | ● | ● | ⊕ | ● | ⊕ | ○ | ○ | ⊕ |
| D4 | ⊕ | ○ | ⊕ |  | ⊗ | ⊗ | ⊗ | ⊗ |
| D5 | ⊕ | ○ | ⊕ | ⊕ | ○ | ⊕ | ⊕ | ○ |
| Raw coverage | 0.37 | 0.52 | 0.57 | 0.61 | 0.58 | 0.34 | 0.70 | 0.75 |
| Unique coverage | 0.10 | 0.26 | 0.12 | 0.16 | 0.35 | 0.11 | 0.09 | 0.14 |
| Consistency | 0.85 | 0.97 | 0.97 | 0.97 | 0.79 | 0.76 | 0.78 | 0.78 |
| Solution coverage | 0.63 | | 0.73 | | 0.69 | | 0.85 | |
| Solution consistency | 0.89 | | 0.84 | | 0.77 | | 0.76 | |

**Remarks:** DMP, Dataset-M Path, Dataset-M replaced all zero-coded values with the variable-specific sample mean computed across valid responses, thereby eliminating potential systematic bias introduced by missing-value markers in set-membership calibration; DAP, Dataset-A Path, Dataset-A retained the original questionnaire coding, treating zero-coded entries (unanswered or non-applicable items) as genuine observations; **●**, Core condition present; ⊗, Core condition absent; ○, Peripheral condition present; ⊕, Peripheral condition absent; blank, condition is irrelevant. Intermediate solutions are reported.; D1, Dual-dimensional perceived physical literacy kernel; D2, Dual-dimensional sense of belonging construction logic; D3, Single-dimensional inclusive leadership openness attribute; D4, Dual identity categorization conduction path; D5, Inter-group comparison output path; D6, State of group‑based social identity under dual‑identity context. Assumption for intermediate solution: D1 is present. Frequency cutoff 2. Core conditions are derived from parsimonious solutions; peripheral conditions derive from intermediate but not parsimonious solutions.
